# Genome-Wide Characterization of PEBP, FD, and GRF Families in *Amomum villosum* Lour. and Their Potential Roles in Flowering

**DOI:** 10.3390/plants15172722

**Published:** 2026-09-05

**Authors:** Ming Lei, Mei Qin, Wei Lin, Jun-Jun He, Shao-Fen Jian, Zhan-Jiang Zhang, Cui Li, Jing Wang

**Affiliations:** 1 Guangxi Key Laboratory of Medicinal Resources Protection and Genetic Improvement, Guangxi Botanical Garden of Medicinal Plants, Nanning 530023, China; leiming@gxyyzwy.com (M.L.); qinmei20210630@163.com (M.Q.); linwei_gbgmp@163.com (W.L.); jsfzc2011@126.com (S.-F.J.); zzj1811@163.com (Z.-J.Z.); 2 National Center for Traditional Chinese Medicine (TCM) Inheritance and Innovation, Guangxi Botanical Garden of Medicinal Plants, Nanning 530023, China; 3 Guangxi Engineering Research Center of TCM Resource Intelligent Creation, Guangxi Botanical Garden of Medicinal Plants, Nanning 530023, China; 4 South Subtropical Crops Research Institute, China Academy of Tropical Agricultural Sciences, Zhanjiang 524013, China; hbj46@catas.cn; 5 Guangdong Dry Farming Water-Saving Agricultural Engineering Technology Research Center, Zhanjiang 524013, China; 6 Guangxi Key Laboratory for High-Quality Formation and Utilization of Dao-di Herbs, Guangxi Botanical Garden of Medicinal Plants, Nanning 530023, China

**Keywords:** *Amomum villosum*, Zingiberaceae, florigen activation complex, *PEBP* gene family, *FD* gene family, *GRF* gene family

## Abstract

A detailed understanding of the molecular mechanisms governing the flowering time of *Amomum villosum* Lour., a medicinal plant within the Zingiberaceae family, is currently lacking. In modern plants, the florigen activation complex (FAC), which includes PEBP, FD/bZIP, and GRF proteins, is known to regulate flowering. In this study, we identified 13 *PEBP*, 5 *FD*, and 19 *GRF* genes within the *A. villosum* genome and conducted phylogenetic, structural and promoter analysis. Notably, cross-species protein–protein interaction predictions and yeast two-hybrid assays uncovered an unexpected interaction pattern: an AREB3-like FD protein (AvFD5) and a GRF protein (AvGRF13) directly interact with specific PEBP members, whereas canonical FD-like proteins (AvFD1 and AvFD4) did not, which contrasts with the classical rice FAC model (Hd3a-14-3-3-OsFD1). These results imply that FAC assembly in *A. villosum* may involve alternative components or regulatory mechanisms, potentially indicating lineage-specific divergence within monocots. This research represents the first systematic characterization of FAC core gene families in *A. villosum* and Zingiberaceae, laying the groundwork for understanding flowering time regulation and facilitating future molecular breeding efforts in this economically significant plant.

## 1. Introduction

*Amomum villosum* Lour., also referred to as *Wurfbainia villosa*, is a perennial herbaceous plant within the genus *Amomum* Roxb. of the Zingiberaceae family [1]. The dried mature fruits of this plant, known as *Amomi Fructus* (AF, or “Sharen” in Chinese), are extensively utilized in traditional Chinese medicine due to their pharmacological properties, which include soothing the fetus, relieving diarrhea, and enhancing appetite [2]. Furthermore, AF is a preferred component in teas, preserved fruits, beverages, liquors, cosmetics, and food additives [3]. Consequently, *A. villosum*, the botanical source of AF, has been recognized by the China Food and Drug Administration as a species with both medicinal and food applications in China [3]. *A. villosum* propagates seedlings through two methods: sexual propagation via seeds and asexual propagation via division. Seedlings propagated asexually by division reach the reproductive growth stage 2–3 years post-planting, whereas those propagated sexually by seeds require 3–4 years to bloom [4]. Therefore, the plant’s extensive growth cycle, coupled with small-scale, decentralized cultivation by individual growers, presents considerable challenges to sustainable industry development [5].

The transition from vegetative to reproductive growth in angiosperms, known as flowering, is a crucial process governed by a complex network of environmental cues, endogenous signals, and genetic programs [6]. Among the various regulatory pathways, the photoperiod pathway is fundamental in regulating flowering time, with its core components being highly conserved across plant species [7]. At the center of this pathway is the florigen activation complex (FAC), which comprises three essential components: FLOWERING LOCUS T (FT)-like proteins, which are members of the phosphatidylethanolamine-binding protein (PEBP) family; FD-like bZIP transcription factors; and 14-3-3 proteins, also referred to as GENERAL REGULATORY FACTORS (GRFs) [8,9,10]. FAC activates downstream floral meristem identity genes such as APETALA 1 and FRUITFULL, thereby initiating floral development [11].

The PEBP family is divided into three primary subclades: FT-like, TERMINAL FLOWER 1 (TFL1)-like, and MOTHER OF FT AND TFL1 (MFT)-like. FT-like proteins facilitate flowering by forming the FAC, whereas TFL1-like proteins function as floral repressors by competing for FAC components to form a florigen repression complex [12,13]. Members of the FD family, which are part of the bZIP transcription factor family, are crucial interaction partners of PEBP proteins and are mainly expressed at the shoot apical meristem [14]. The 14-3-3 (GRF) proteins act as highly conserved scaffolding proteins that bridge FT and FD, aiding in the formation of the FAC [10]. Within this complex, FD is phosphorylated at its C-terminal SAP motif by calcium-dependent protein kinases, allowing for 14-3-3 binding, whereas the C-terminal region of 14-3-3 interacts with FT [15,16].

Recent transcriptomic analyses have been utilized to explore the flowering mechanism of *A. villosum*. Zhu et al. conducted RNA-seq on rhizomes, stems, and leaves of *A. villosum* during both the vegetative and flowering stages, identifying 3061 differentially expressed genes and 43 key genes associated with flowering [17]. Another study reported the existence of distinct early flowering *A. villosum* plants capable of flowering and fruiting within their first year of planting [5]. Transcriptomic analysis identified an *FD* homologue gene, *AvFD1*, which is highly expressed in stolon tips and flower buds, indicating its potential role as a positive regulator of flowering initiation [5,18]. These studies have provided valuable transcriptomic resources and preliminary molecular insights into the regulation of flowering in this species. Nevertheless, a comprehensive genome-wide identification and characterization of the core FAC component gene families—*PEBP*, *FD/bZIP*, and *GRF*—has not yet been conducted in *A. villosum*. This gap hinders the understanding of the molecular mechanism of flowering and, consequently, the breeding of early flowering and high-yielding *A. villosum* varieties.

In this study, we systematically identified and characterized the *PEBP*, *FD*, and *GRF* gene families in the *A. villosum* genome. Our analyses encompassed their physicochemical properties, chromosomal localization, phylogenetic relationships, gene duplication events, conserved motifs, gene structures, and promoter cis-regulatory elements. Furthermore, we predicted protein–protein interactions (PPIs) among PEBP, FD, and GRF proteins through cross-species PPI analysis and validated the selected interactions using yeast two-hybrid (Y2H) assays. Our findings revealed that an ABA-responsive element binding protein 3 (AREB3)-like FD protein (AvFD5) and a GRF protein (AvGRF13) directly interact with specific PEBP members in *A. villosum*, whereas the canonical FD-like proteins (AvFD1 and AvFD4) exhibited no detectable interactions. This interaction pattern diverges from that of classical rice FAC model (Hd3a-14-3-3-OsFD1). In summary, this study provides a comprehensive foundation for understanding the molecular mechanisms by which FAC regulate flowering time in *A. villosum* and offers valuable resources for future functional studies on this medicinal plant.

## 2. Results

### 2.1. Identification of AvPEBPs, AvFDs, and AvGRFs in A. villosum

**Figure 1:**
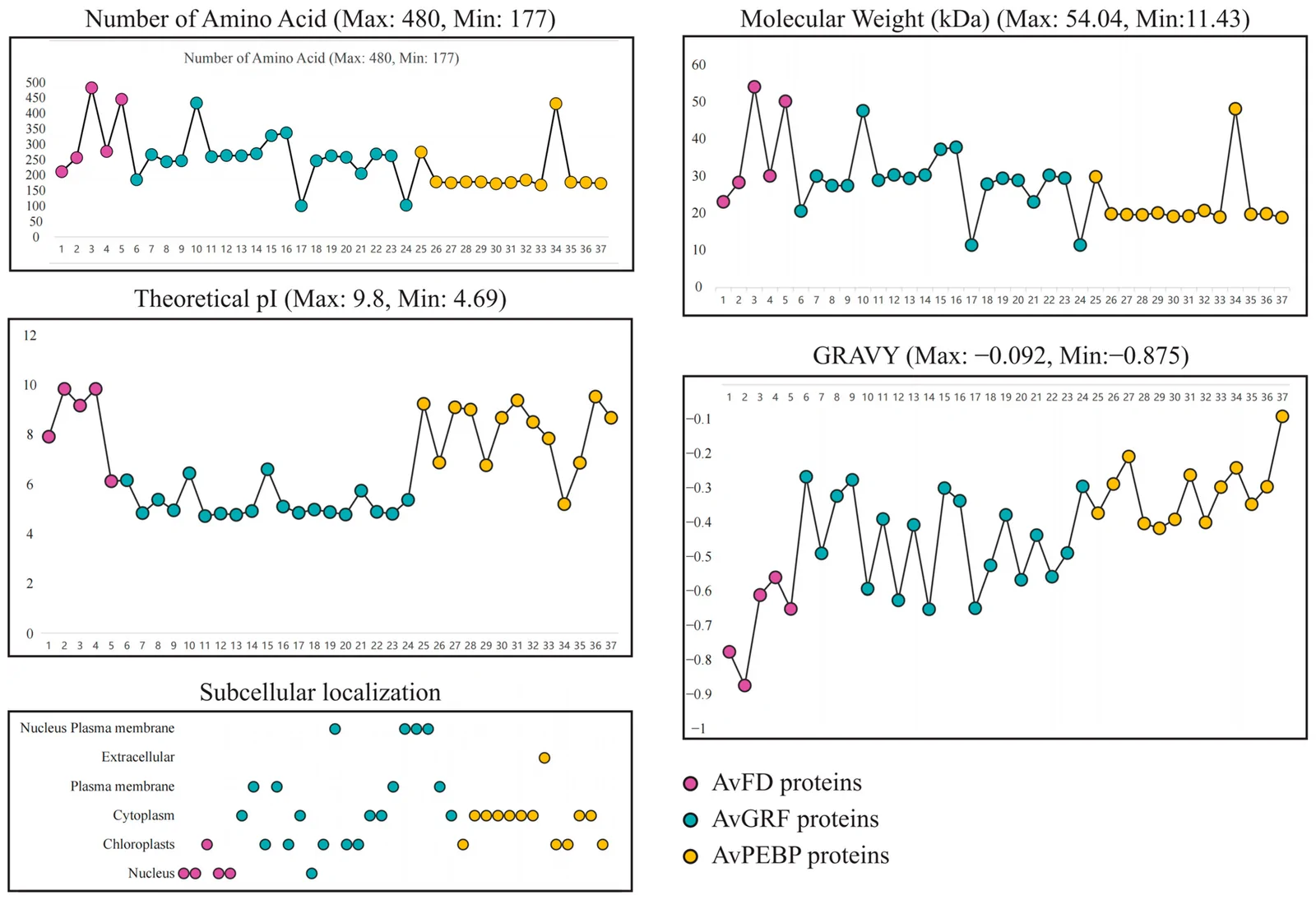
Physicochemical properties of the 37 identified AvPEBP, AvFD, and AvGRF proteins. The AvPEBP proteins are indicated in yellow, the AvFD proteins in pink, and the AvGRF proteins in blue.

Through the integration of the Hidden Markov Model (HMM) and BLAST v2.14.1+ searches, all potential members of the *AvPEBP*, *AvFD*, and *AvGRF* gene families were identified within the *A. villosum* genome. Following the elimination of redundancies, 13 *AvPEBP*, 5 *AvFD*, and 19 *AvGRF* genes were identified. Each family member was subsequently named according to its chromosomal location. The 37 genes were analyzed for amino acid (aa) sequence length, protein isoelectric point (pI), molecular weight (MW), and subcellular localization (Figure 1, Table S1). The results showed that the three gene families in *A. villosum* possess distinct yet characteristic physicochemical properties.

#### 2.1.1. AvPEBP Family

Thirteen AvPEBP proteins were identified in the *A. villosum* genome, Table S1 and Figure 1 illustrate that the length of these proteins ranged from 167 aa for AvPEBP9 to 429 aa for AvPEBP10, with an average length of 198 aa. Notably, AvPEBP10, at 429 aa, was considerably longer than the other proteins, which predominantly ranged from 167 to 183 aa, except for AvPEBP1, which was 273 aa long. The MW of AvPEBPs varied from 18.86 kDa (AvPEBP13) to 48.13 kDa (AvPEBP10), with an average MW of 22.09 kDa. The pI ranged from 5.17 (AvPEBP10) to 9.49 (AvPEBP12), with an average of 7.96. Among these proteins, eight were categorized as basic proteins (pI > 8.00: AvPEBP1, AvPEBP3, AvPEBP4, AvPEBP6, AvPEBP7, AvPEBP8, AvPEBP12, and AvPEBP13), four as neutral or weakly acidic (pI 6.73–7.81: AvPEBP2, AvPEBP5, AvPEBP9, and AvPEBP11), and one as acidic (AvPEBP10, pI = 5.17). All AvPEBP proteins demonstrated negative Grand Average of Hydropathy (GRAVY) values, indicating their hydrophilic properties. In terms of subcellular localization, seven AvPEBP proteins were found in the cytoplasm, five in chloroplasts, and one in the extracellular space.

#### 2.1.2. AvFD Family

Following the exclusion of redundant transcripts and potential pseudogenes that lacked either the bZIP domain or the SAP motif (L[X]-R[QL]-R[H]-X-X-S[T]-A[GCTM]-P[ISQE]) in the C-terminal region, five members of the *AvFD* gene family were identified within the *A. villosum* genome. As illustrated in Figure 1, these five AvFD proteins exhibited a length range from 210 aa (AvFD1) to 480 aa (AvFD3), with an average of 333 aa. Their MW varied from 23.08 kDa (AvFD1) to 54.04 kDa (AvFD3), with an average of 37.13 kDa. The pI values ranged from 6.09 (AvFD5) to 9.80 (AvFD2 and AvFD4), with an average pI of 8.52. Among these proteins, three were classified as basic (pI > 8.00: AvFD2, AvFD3, and AvFD4), whereas two were neutral or weakly acidic (AvFD1: pI = 7.88; AvFD5: pI = 6.09). Consistent with AvPEBPs, all AvFD proteins demonstrated hydrophilic properties, as indicated by negative GRAVY values. Subcellular localization analysis indicated that four AvFD proteins were localized to the nucleus, whereas one was localized to chloroplasts (AvFD3).

#### 2.1.3. AvGRF Family

The 19 AvGRF proteins exhibited lengths ranging from 100 aa for AvGRF12 to 431 aa for AvGRF5, with a mean length of 245 aa. Their MW varied from 11.43 kDa for AvGRF12 to 47.60 kDa for AvGRF5, averaging 29.15 kDa (Table S1). The theoretical pI values of AvGRF proteins ranged from 4.69 for AvGRF6 to 6.57 for AvGRF10, with an average pI of 5.15 (Figure 1, Table S1). All 19 AvGRF members were classified as neutral. Furthermore, predictions indicated that all AvGRF proteins are hydrophilic, as evidenced by their negative GRAVY values. Regarding subcellular localization, seven AvGRF proteins were found in the chloroplasts, six in the plasma membrane, five in the cytoplasm, and one in the nucleus; notably, four proteins exhibited dual localization in both the nucleus and plasma membrane (Figure 1, Table S1).

### 2.2. Chromosomal Localization and Collinearity Analysis of AvPEBP, AvFD, and AvGRF Genes

**Figure 2:**
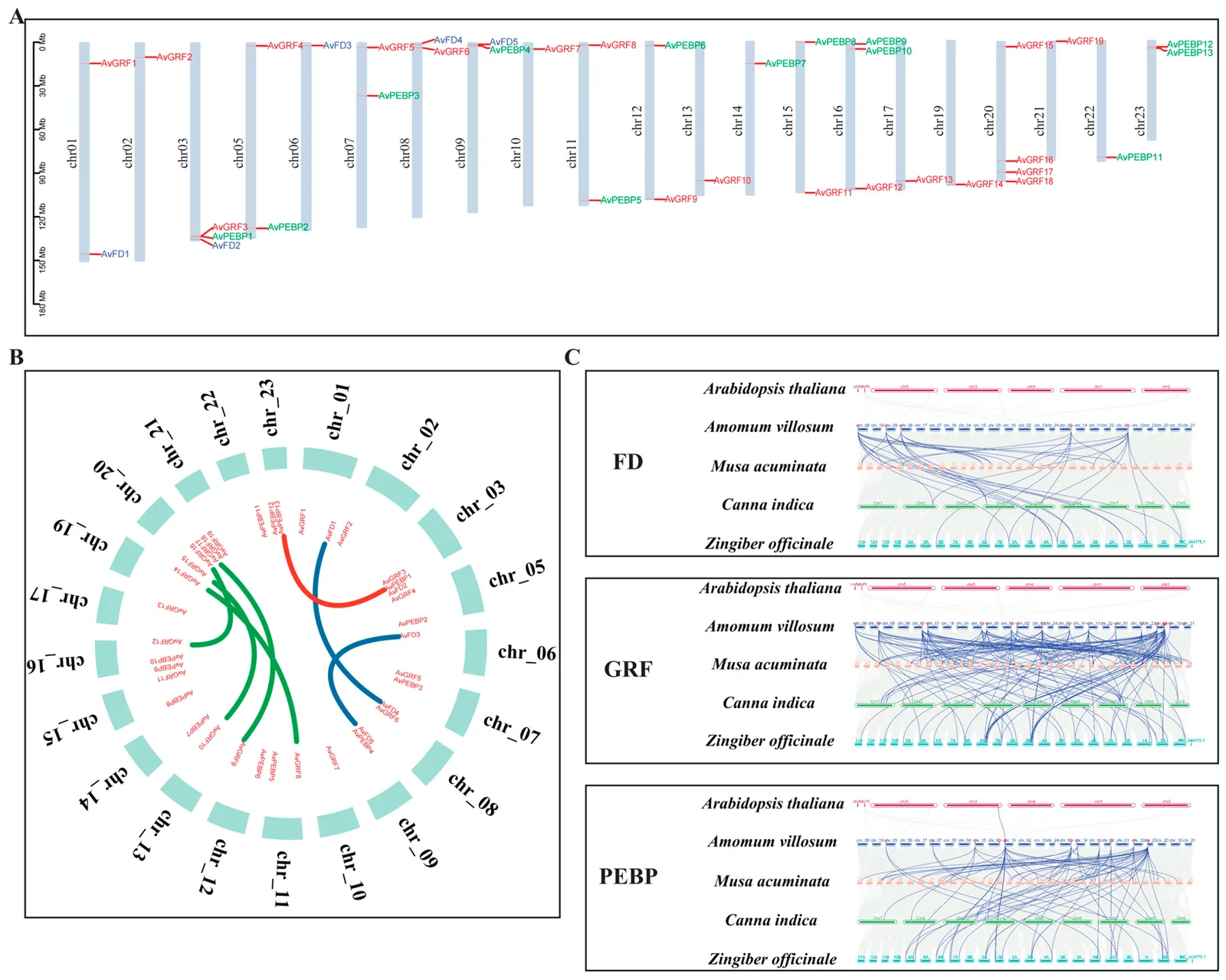
Chromosomal localization and collinearity analyses of *AvPEBP*, *AvFD*, and *AvGRF* genes in *A. villosum*. (**A**) The chromosomal distribution of the 37 identified genes across 21 chromosomes; (**B**) The intra-species collinearity relationships among the three gene families; (**C**) Inter-species collinearity analysis between *A. villosum* and four reference species (*Arabidopsis thaliana*, *Musa acuminata*, *Canna indica*, and *Zingiber officinale*).

To examine the chromosomal distribution of the identified *AvPEBP*, *AvFD*, and *AvGRF* genes in *A. villosum*, all 37 genes were mapped onto the chromosomes using the genome annotation. As illustrated in Figure 2, these genes were distributed across 21 chromosomes. Among the three families, the *AvGRF* family exhibited the most extensive distribution, with 19 members located on 15 chromosomes in the genome. The *AvPEBP* family, comprising 13 members, was distributed across 11 chromosomes. The *AvFD* family, consisting of five members, was distributed across five chromosomes (Figure 2A).

Intra-species collinearity analysis revealed that 19 *AvGRF*s participated in four pairs of segmental duplications: *AvGRF8*/*AvGRF14*, *AvGRF9*/*AvGRF17*, *AvGRF10*/*AvGRF15*, and *AvGRF12*/*AvGRF16*. Phylogenetic classification indicated that, with the exception of the *AvGRF10*/*AvGRF15* pair, which is categorized under Group II (minor clade), the other three pairs of segmentally duplicated genes are classified under Group I (major clade). Furthermore, two collinear relationships were detected within the *AvFD* family (*AvFD1*/*AvFD4*, *AvFD3*/*AvFD5*), and one collinear pair was identified in the *AvPEBP* family (*AvPEBP1*/*AvPEBP13*) (Figure 2B).

To elucidate the evolutionary trajectory and origin of the *AvPEBP*, *AvFD*, and *AvGRF* family members, collinearity maps were constructed by comparing *A. villosum* with *Arabidopsis thaliana* and three monocotyledonous plants: *Musa acuminata*, *Canna indica*, and *Zingiber officinale* (Figure 2C). The *AvPEBP*s displayed 33, 9, and 38 homologous gene pairs with *M. acuminata*, *C. indica*, and *Z. officinale*, respectively, which is significantly higher than the single homologous gene pairs identified between *A. villosum* and *A. thaliana*. Similarly, *AvFD*s exhibited 22, 5, and 19 homologous gene pairs, whereas *AvGRF*s showed 81, 21, and 54 homologous gene pairs with *M. acuminata*, *C. indica*, and *Z. officinale*, respectively. No collinearity was detected among *AvFD* and *AvGRF* family genes in *A. thaliana*. This pattern can be attributed to the fact that *A. villosum*, *M. acuminata*, *C. indica*, and *Z. officinale* all belong to the order Zingiberales, indicating a closer evolutionary relationship with other species within the same order. Additionally, as a monocotyledonous plant, *A. villosum* exhibits a relatively distant evolutionary relationship with dicotyledonous species such as *A. thaliana*.

### 2.3. Phylogenetic Analysis of AvPEBP, AvFD, and AvGRF Families in A. villosum

**Figure 3:**
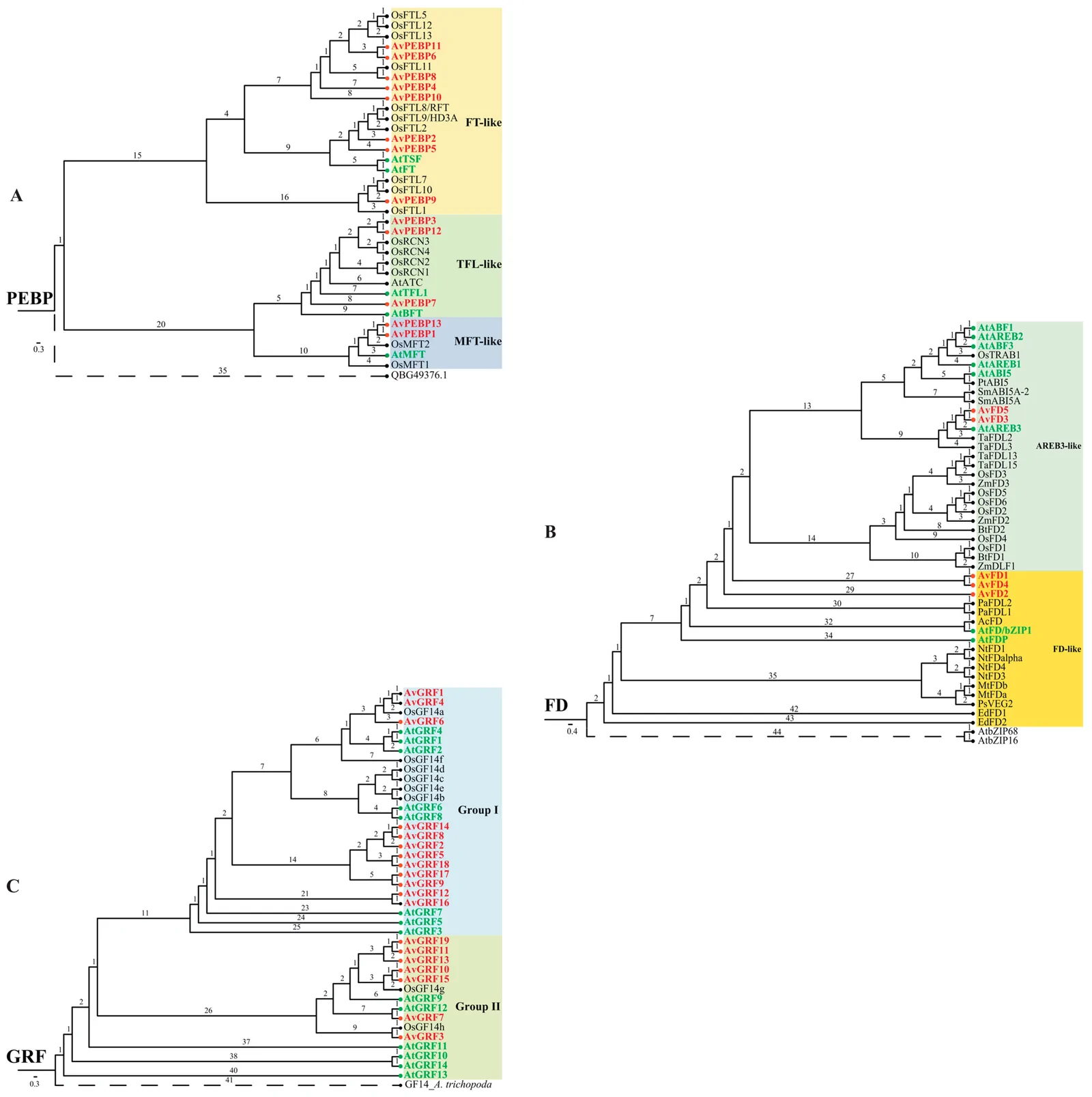
RAxML-based phylogenetic trees of AvPEBP, AvFD, and AvGRF families in *A. villosum*. The red solid dots represent proteins derived from *A. villosum*. (**A**) AvPEBP family; (**B**) AvFD family; (**C**) AvGRF family.

Phylogenetic trees were generated using three distinct methodologies: Neighbor-Joining (NJ), Bayesian Inference (BI), and RAxML. The topological structures exhibited substantial congruence among the three methods. Figure 3 illustrates the RAxML tree, while the NJ and BI trees are depicted in Supplementary Figures S1–S6.

#### 2.3.1. AvPEBP Family

The gymnosperm *Ginkgo biloba* (QBG49376.1) was used as the outgroup. Reference sequences from *A. thaliana* (AtFT, AtTSF, AtTFL1, AtATC, AtBFT, and AtMFT) and *Oryza sativa* (OsFTL2/5/8/9/11/12/13, OsMFT1/2, and RCN1–4) were incorporated. As illustrated in Figure 3A, all 13 AvPEBP sequences were consistently categorized into three primary clades.

The FT-like clade comprised eight AvPEBP members: AvPEBP2, AvPEBP4, AvPEBP5, AvPEBP6, AvPEBP8, AvPEBP9, AvPEBP10, and AvPEBP11. These sequences were grouped with AtFT/AtTSF and OsFTL2/5/8/9/11/12/13. Within this clade, monocot (AvPEBP and OsFTLs) and dicot (AtFT/AtTSF) sequences were intermixed, and did not form distinct subclades. The branch support values for this clade were as follows: The NJ bootstrap values varied from 10 to 90, BI posterior probabilities were 1.00 for most internal branches, and RAxML bootstrap values ranged from 25 to 99.

The TFL1-like clade comprised three AvPEBP members: AvPEBP3, AvPEBP7, and AvPEBP12. These members clustered with AtTFL1, AtATC, AtBFT, and rice RCN1–4. The NJ bootstrap values for this clade varied from 2 to 74, whereas the BI posterior probabilities were 1.00 for most branches.

The MFT-like clade included two AvPEBP members, AvPEBP1 and AvPEBP13, which clustered with AtMFT and OsMFT1/OsMFT2. The NJ bootstrap values for this clade ranged from 32 to 89, and the BI posterior probabilities ranged from 0.94 to 1.00.

#### 2.3.2. AvFD Family

FD, FD PARALOGUE (FDP), and AREB3 are members of group A of the bZIP transcription factor family [14]. FD and FDP are two closely related paralogs that have evolved distinct roles in the regulation of flowering, whereas AREB3 is part of the older ABI5/AREB/ABF subclade, primarily associated with abscisic acid signaling. However, it also functions redundantly with FD in promoting flowering [19,20]. To further investigate the evolutionary relationships among AvFD members and their orthologs in green plants, we conducted a comprehensive phylogenetic analysis using sequences from a diverse array of species. AtbZIP68 and AtbZIP16 sequences served as outgroups. The study incorporated AvFD family sequences from various species, including monocots such as rice, wheat, maize, bamboo, and *A. villosum*; dicots such as pea, alfalfa, London plane, tobacco, kiwifruit, wild loquat, and citrus; gymnosperms such as loblolly pine and white spruce; and a fern, *Selaginella moellendorffii*. As illustrated in Figure 3B, all sequences were systematically categorized into two primary clades.

The FD-like clade comprised AvFD1, AvFD2, and AvFD4, which were grouped with the canonical FD/FDP members. These included AtFD/bZIP1, AtFDP, and other dicot FD homologs such as MtFDa/b, PsVEG2, NtFD1/3/4/α, EdFD1/2, PaFDL1/2, and AvFD.

The AREB3-like clade consisted of AvFD3 and AvFD5, which were associated with AREB/ABF/ABI5-type transcription factors. These factors were involved in ABA signaling and stress responses and included AtAREB3, AtABI5, AtAREB1/2/3, AtABF1/3, OsTRAB1, PtABI5, and SmABI5A.

#### 2.3.3. AvGRF Family

The 14-3-3 protein from *Amborella trichopoda* (GF14ε) was used as the outgroup. Reference sequences from *A. thaliana* (AtGRF1–14) and *O. sativa* (OsGF14a–h) were incorporated. As illustrated in Figure 3C, all 19 AvGRF sequences were systematically categorized into two primary clades, aligning with the findings of a previous comparative genomic study in plants [21].

Group I (major clade) comprised 12 AvGRF members: AvGRF1, AvGRF2, AvGRF4, AvGRF5, AvGRF6, AvGRF8, AvGRF9, AvGRF12, AvGRF14, AvGRF16, AvGRF17, and AvGRF18. These sequences clustered with the majority of Group I AtGRF homologs (AtGRF1–8, AtGRF12) and OsGF14a/OsGF14f. Within this clade, AvGRF2, AvGRF8, and AvGRF14 formed a subclade with AtGRF7 (GRF7/14-3-3v). The branch support values for this clade were as follows: NJ bootstrap values ranged from 5 to 100, BI posterior probabilities were 1.00 for most major branches, and RAxML bootstrap values ranged from 1 to 98.

Group II (minor clade) comprised the remaining seven AvGRF members: AvGRF3, AvGRF7, AvGRF10, AvGRF11, AvGRF13, AvGRF15, and AvGRF19. These sequences were associated with AtGRF9, AtGRF10, AtGRF11, and AtGRF14 (members of Group II in *Arabidopsis*), as well as OsGF14g/OsGF14h. The NJ bootstrap values for this clade ranged from 11 to 85, whereas the RAxML bootstrap values varied from 18 to 82.

### 2.4. Phylogenetic Classification and Structural Features of the AvPEBP, AvFD, and AvGRF Families

**Figure 4:**
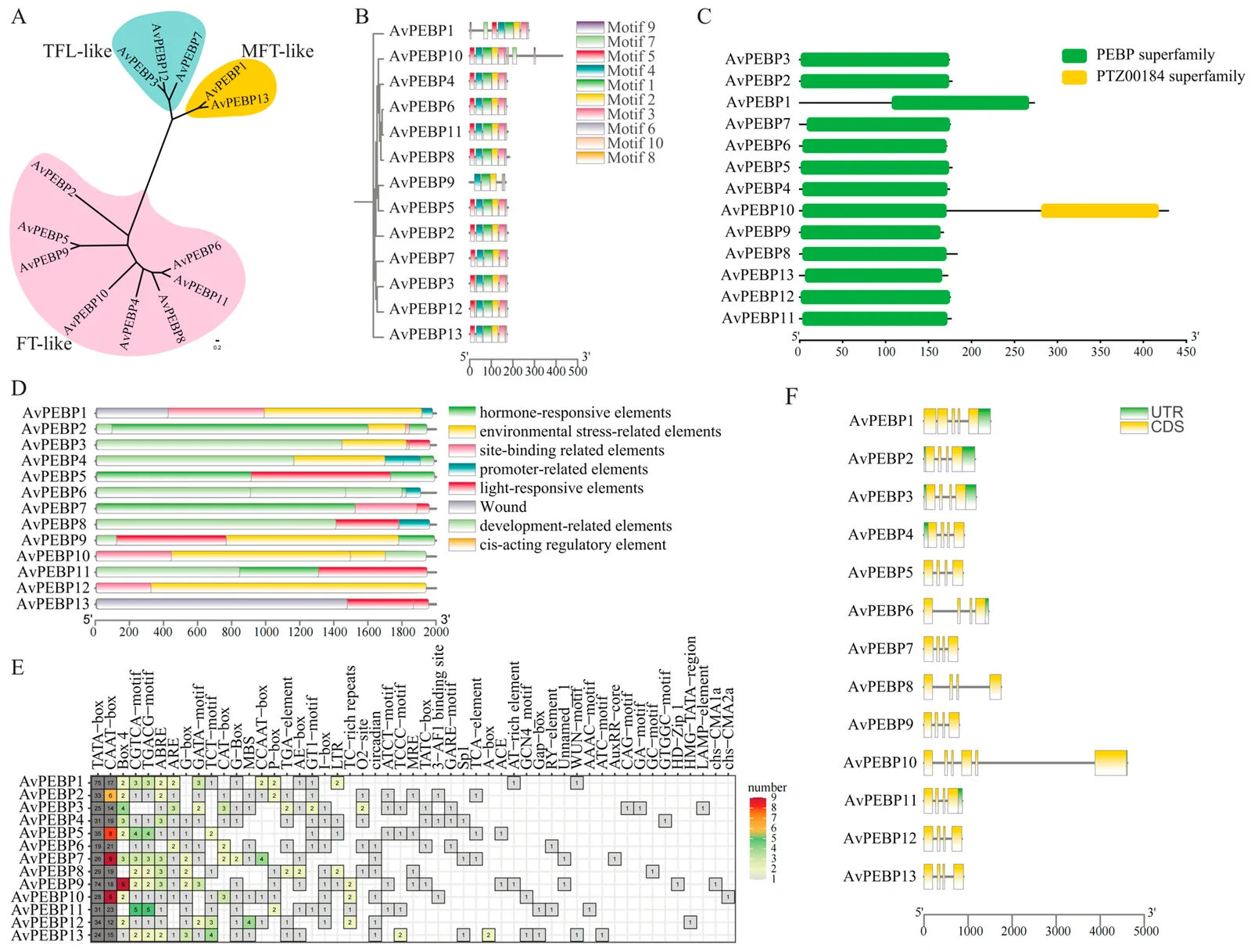
Phylogenetic relationships and structural characterization of AvPEBP members in *A. villosum*. (**A**) Phylogenetic tree of AvPEBP proteins from *A. villosum*; (**B**) phylogenetic tree of AvPEBP proteins annotated with conserved motifs; (**C**) conserved domain architecture of AvPEBP proteins; (**D**) distribution of predicted cis-regulatory elements in *AvPEBP* promoter regions; (**E**) distribution of cis-regulatory element categories in *AvPEBP* genes; (**F**) gene structure, specifically exon–intron organization of *AvPEBP* genes.

**Figure 5:**
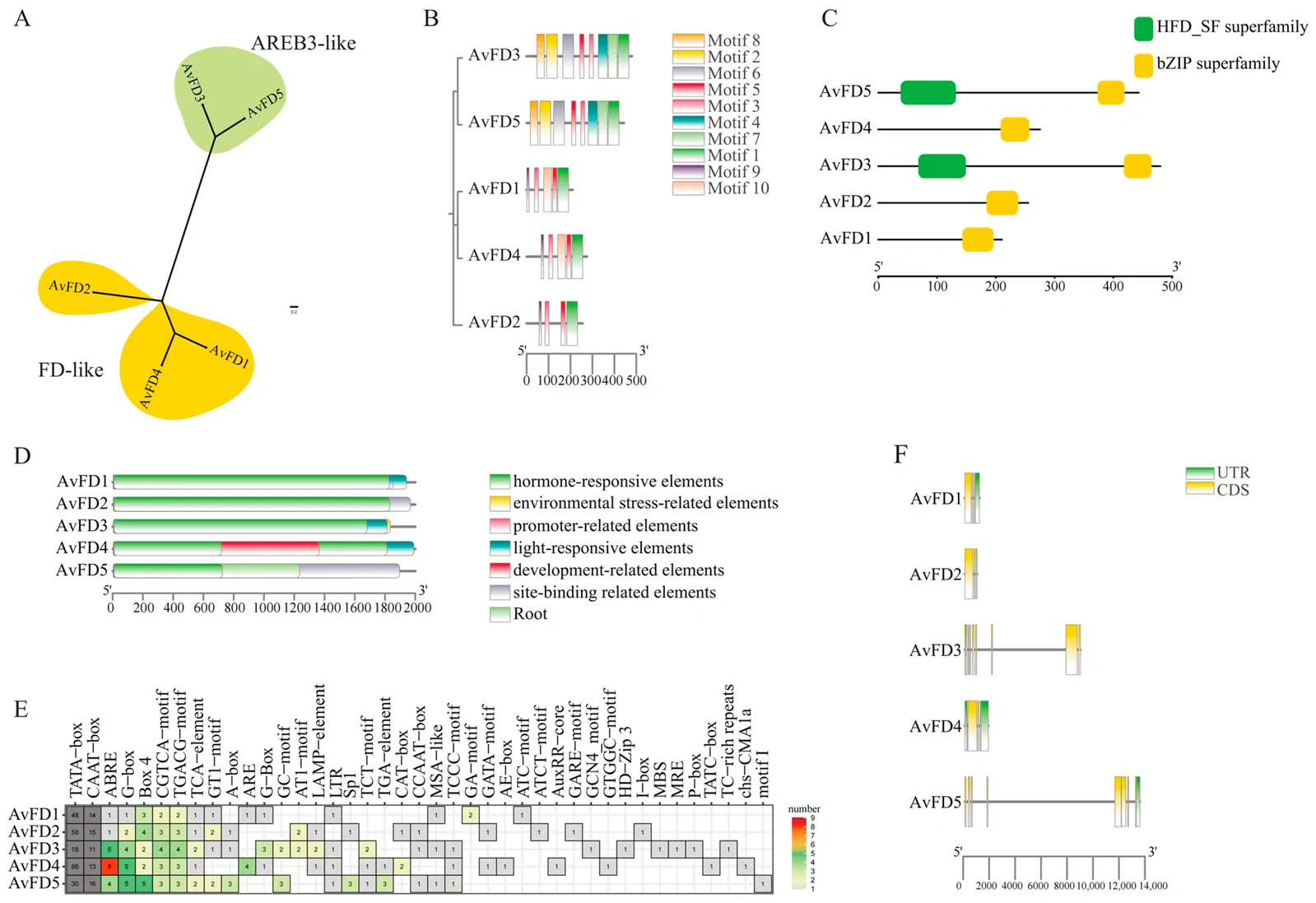
Phylogenetic relationships and domain architecture of Av*FD* members in *A. villosum*. (**A**) Phylogenetic tree of AvFD proteins; (**B**) phylogenetic tree of AvFD proteins annotated with conserved motifs; (**C**) conserved domain architecture of AvFD proteins; (**D**) the distribution of predicted cis-regulatory elements in *AvFD* promoter regions; (**E**) distribution of cis-regulatory element categories in *AvFD* genes; (**F**) gene structure, specifically exon–intron organization of *AvFD* genes.

**Figure 6:**
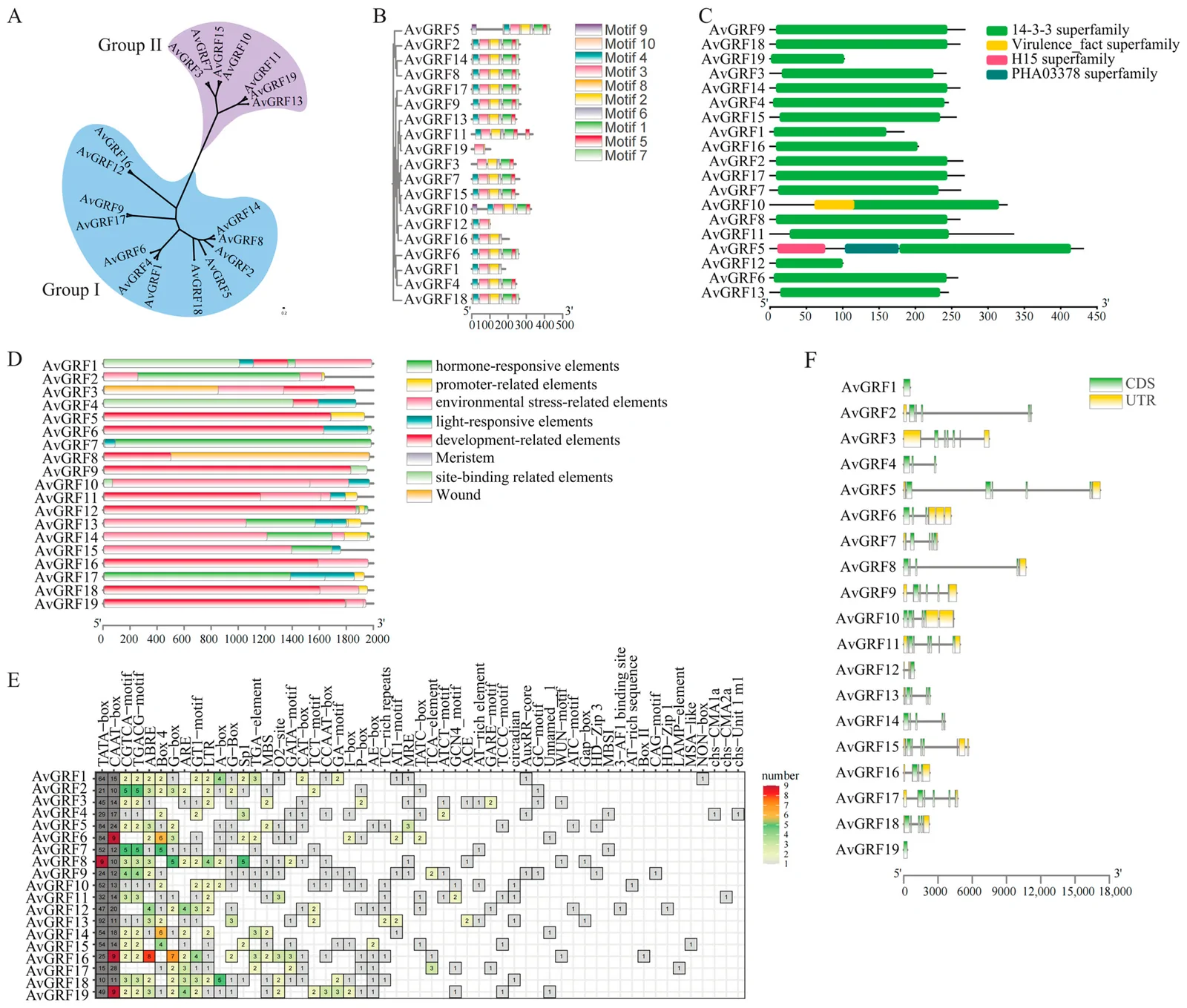
Phylogenetic relationships and structural characterization of AvGRF members in *A. villosum*. (**A**) Phylogenetic tree of AvGRF proteins; (**B**) phylogenetic tree of AvGRF proteins annotated with conserved motifs; (**C**) conserved domain architecture of AvGRF proteins; (**D**) the distribution of predicted cis-regulatory elements in *AvGRF* promoter regions; (**E**) distribution of cis-regulatory element categories in *AvGRF* genes; (**F**) gene structure, specifically exon–intron organization, of *AvGRF* genes.

To elucidate the evolutionary relationships and structural diversification among the three FAC core families, we performed phylogenetic reconstruction and systematically analyzed their conserved motifs, domain architectures, exon–intron organizations, and promoter cis-regulatory elements (Figure 4, Figure 5 and Figure 6; Table S2).

#### 2.4.1. Phylogenetic Classification

RAxML-based phylogenetic trees, which were largely congruent with NJ and BI topologies (Figure 3 and Figures S1–S6), classified the 13 AvPEBP members into three conserved subfamilies: the FT-like clade (eight members: AvPEBP2, AvPEBP4, AvPEBP5, AvPEBP6, AvPEBP8, AvPEBP9, AvPEBP10, and AvPEBP11), the TFL1-like clade (three members: AvPEBP3, AvPEBP7, and AvPEBP12), and the MFT-like clade (two members: AvPEBP1 and AvPEBP13) (Figure 3A). For the five AvFD members, the tree resolved two major clades: the canonical FD-like clade (AvFD1, AvFD2, and AvFD4, clustered with AtFD/AtFDP and monocot FD homologs) and the AREB3-like clade (AvFD3 and AvFD5, associated with ABA-responsive element-binding factors) (Figure 3B). The 19 AvGRF proteins were partitioned into Group I (12 members: AvGRF1, AvGRF2, AvGRF4, AvGRF5, AvGRF6, AvGRF8, AvGRF9, AvGRF12, AvGRF14, AvGRF16, AvGRF17, and AvGRF18) and Group II (seven members: AvGRF3, AvGRF7, AvGRF10, AvGRF11, AvGRF13, AvGRF15, and AvGRF19), aligning with the bipartite classification established in other angiosperms (Figure 3C).

#### 2.4.2. Conserved Motifs and Domain Architectures

Despite the conserved core domains across all families, motif composition and auxiliary domain presence varied in a clade-specific manner. All AvPEBP members uniformly possessed a complete set of ten motifs (Motifs 1–10) and the core PEBP superfamily domain, with the sole exception of AvPEBP10, which contained an additional PTZ00184 extension at its C-terminus (Figure 4B,C). In contrast, the AvFD family exhibited pronounced motif reduction between clades: the AREB3-like members (AvFD3 and AvFD5) retained Motifs 1–8, whereas the FD-like (AvFD1 and AvFD4) and FDP-like (AvFD2) members progressively lacked Motifs 2, 4, 6, 7, 8, and 10 (Figure 5B). Domain annotation further revealed that only the AREB3-like AvFD3 and AvFD5 possessed an additional HFD_SF domain alongside the core bZIP domain, implying their potential functional specialization beyond the canonical flowering regulation (Figure 5C). Within the AvGRF family, Group I members generally preserved all ten motifs, whereas Group II members frequently lacked specific motifs such as Motif 2 or Motif 8 (Figure 6B). All AvGRF proteins shared the 14-3-3 superfamily domain, except for AvGRF5 and AvGRF10, which carried additional H15 and Virulence_fact domains, respectively (Figure 6C).

#### 2.4.3. Gene Structures and Promoter Landscapes

Exon–intron organization was highly variable both within and across families: *AvPEBP* genes contained 4–6 exons (with the six-exon *AvPEBP10* being the longest), *AvFD* genes ranged from 3 to 8 exons (the AREB3-like genes uniquely harboring eight exons), and *AvGRF* genes displayed the widest range from 1 to 7 exons (Figure 4F and Figure 6F). Despite these structural differences, promoter analysis revealed a shared regulatory landscape across all three families: the CAAT-box and TATA-box (core promoter elements), light-responsive elements (Box 4 and G-box), and hormone-responsive elements (ABRE for ABA and CGTCA-motif/TGACG-motif for MeJA) were ubiquitously enriched in the majority of gene promoters (Figure 4D,E, Figure 5D,E and Figure 6D,E; Table S2). Notably, clade-specific enrichments were also observed; for instance, ABRE elements were more densely distributed in the promoters of AREB3-like *AvFD* genes (*AvFD3* and *AvFD5*) than in FD-like members, consistent with their phylogenetic affinity to stress-responsive bZIP factors. Comprehensive cis-element distributions and their functional categories are listed in Supplementary Table S2.

### 2.5. Tissue-Specific Expression Patterns of the AvPEBP, AvFD, and AvGRF Gene Families in A. villosum

**Figure 7:**
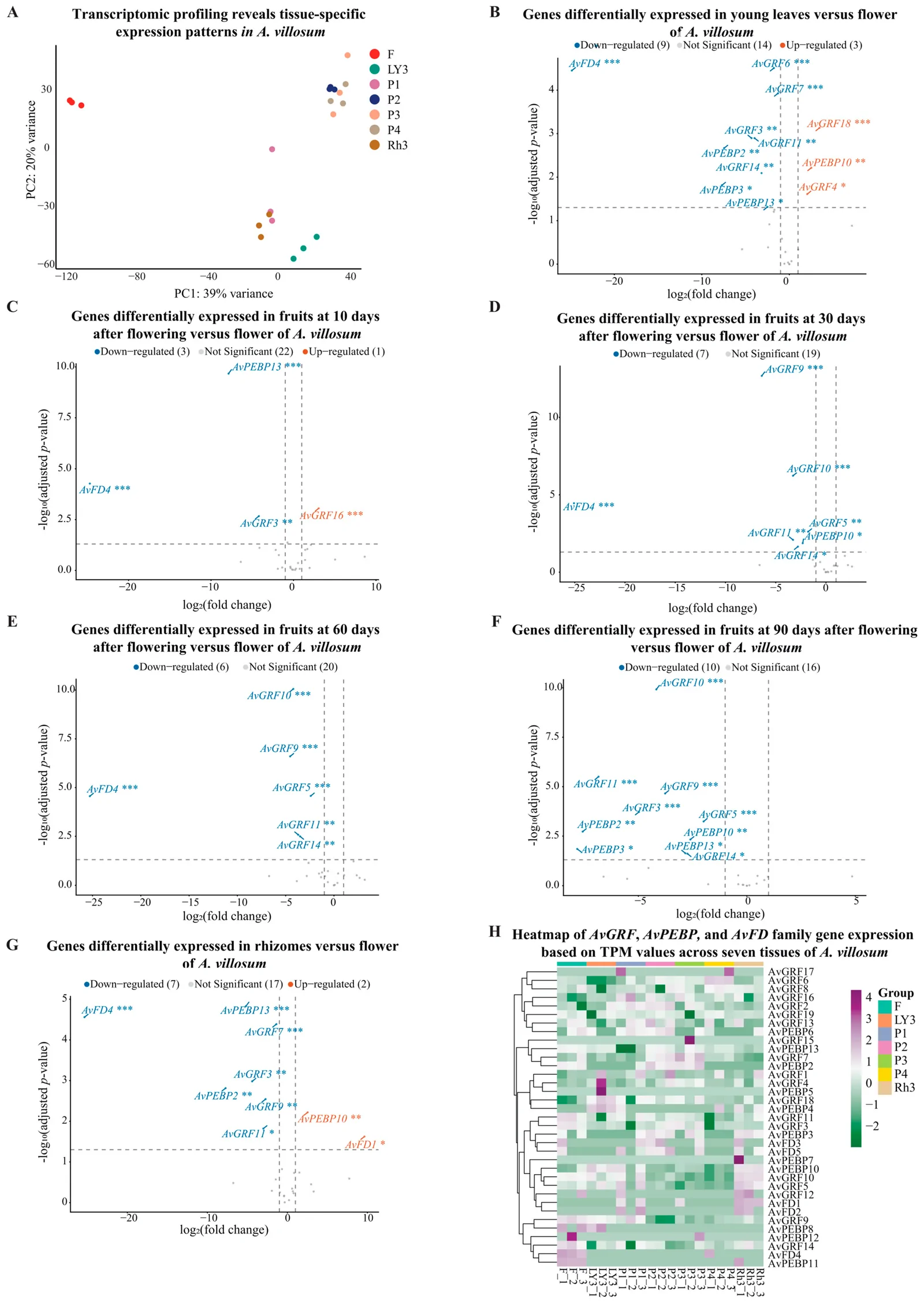
Transcriptomic profiling reveals tissue-specific expression patterns across seven *A. villosum* tissues. (**A**) Principal component analysis (PCA) of transcriptomic data from seven *A. villosum* tissues. PC1 and PC2 explained 39% and 20% of the total variances, respectively. Samples are color-coded by tissue type: F (flowers), LY3 (young leaves), Rh3 (rhizomes), and P1–P4 (fruits at 10, 30, 60, and 90 days after flowering). (**B**–**G**) Volcano plots of differentially expressed genes (DEGs) between each of the six tissues and flowers (**F**). DEGs were defined as genes with $|{log}_{2}(fold\;change)|\geq 1$ and adjusted *p* < 0.05. Significantly upregulated and downregulated genes are shown in red and blue, respectively; genes with no significant differences are shown in gray. Selected target genes from the *AvPEBP*, *AvFD*, and *AvGRF* families are labeled. (**B**) LY3 vs. F; (**C**) Rh3 vs. F; (**D**) P1 vs. F; (**E**) P2 vs. F; (**F**) P3 vs. F; (**G**) P4 vs. F. (**H**) Heatmap showing the expression patterns of 35 target genes from the *AvPEBP*, *AvFD*, and *AvGRF* families across seven tissues of *A. villosum* (*AvPEBP1* and *AvPEBP9* were not included due to undetectable expression across all samples). Expression levels were calculated as transcripts per million (TPM), ${log}_{2}(TPM+1)$-transformed, and row-scaled Z-scores. The color scale from green to purple indicates low to high relative expression levels. Rows represent individual genes, and columns represent biological replicates from each tissue. Genes and samples were hierarchically clustered based on expression similarity. Tissues are indicated by colored bars at the top: F (flower), LY3 (young leaf), Rh3 (rhizome), and P1–P4 (fruits at 10, 30, 60, and 90 days after flowering). (**H**) Hierarchical clustering heatmap showing the expression patterns of 35 AvPEBP, AvFD, and AvGRF genes across seven tissues. Significance was determined using adjusted *p*-values from DESeq2 analysis. Asterisks indicate significant differences: * *p* < 0.05, ** *p* < 0.01, *** *p* < 0.001.

To investigate the tissue-specific expression patterns of *AvPEBP*s, *AvFD*s, and *AvGRF*s across various developmental stages and organs, transcriptome analysis was performed on seven distinct tissues of *A. villosum*, including flowers (F), young leaves (LY3), rhizomes (Rh3), and four fruit developmental stages, namely fruits at 10 days (P1), 30 days (P2), 60 days (P3), and 90 days (P4) after flowering (Table S7). Principal component analysis (PCA) revealed that the seven tissue types were clearly separated into distinct clusters, with F, LY3, and Rh3 exhibiting the most pronounced separation along the PC1 axis. This separation indicates significant differences in gene expression profiles among these tissues (Figure 7A).

To further explore the differential expression patterns of these gene families across various tissues, pairwise differential expression analyses were performed using F as the reference control against LY3, Rh3, and four fruit developmental stages (P1, P2, P3, and P4). Before performing the differential expression analysis, genes exhibiting low expression levels (counts < 10 in at least three samples) were excluded, resulting in the retention of 26 of the 37 target genes for subsequent analyses (Table S8).

The results showed that LY3 exhibited the highest number of differentially expressed genes (DEGs) relative to F, with a total of 12 DEGs, comprising three upregulated and nine downregulated genes. This was followed by P4, which exhibited 10 DEGs, all of which were downregulated. Rh3 presented nine DEGs, including two upregulated and seven downregulated. P2 had 7 DEGs, all downregulated, and P3 had six DEGs, also all downregulated. In contrast, P1 exhibited the fewest DEGs, with four DEGs, consisting of one upregulated and three downregulated genes (Figure 7B–G, Table S9).

At the level of individual genes within the FD family, *AvFD4* exhibited significant downregulation in LY3, Rh3, P1, P2, and P3 $\left({log}_{2}FC>-20\right)$, while no significant difference was observed in P4. Conversely, *AvFD1* was significantly upregulated in Rh3, with no significant differences detected in other tissues (Table S6). In the GRF family, *AvGRF11* was markedly downregulated in LY3, Rh3, P2, P3, and P4 $({log}_{2}FC$ range: −3.01 to −6.84), with P1 showing no significant change. AvGRF3 was significantly downregulated in LY3, Rh3, P1, and P4 (${log}_{2}FC$ range: −4.20 to −4.97), but no significant differences were noted in P2 and P3. Additionally, *AvGRF9*, *AvGRF10*, *AvGRF14*, and *AvGRF5* were significantly downregulated in P2, P3, and P4, while *AvGRF7* was downregulated only in LY3 and Rh3, and *AvGRF6* only in LY3. Regarding upregulated genes, *AvGRF18* and *AvGRF4* were upregulated in LY3, *AvGRF16* in P1, *AvFD1* in Rh3, and *AvPEBP10* in both LY3 and Rh3. Within the PEBP family, *AvPEBP10* was significantly upregulated in LY3 and Rh3, significantly downregulated in P2 and P4, and showed no significant differences in P1 and P3. *AvPEBP13* was significantly downregulated in LY3, P1, and P4, with no significant differences in P2 and P3. *AvPEBP2* was significantly downregulated in LY3 and P4, with no significant differences in other fruit stages, and *AvPEBP3* was significantly downregulated in LY3 and P4, with no significant differences in other tissues.

To facilitate the visualization and comparison of expression dynamics of the three gene families, a hierarchical clustering analysis was performed based on the TPM values of the 35 target genes (Table S8). An expression heatmap was subsequently generated (Figure 7H). Notably, *AvPEBP1* and *AvPEBP9* were excluded due to their undetectable expression across all samples. The analysis resulted in the classification of the 35 genes into six primary clusters: Cluster 1, comprising *AvGRF9*, *AvPEBP8*, *AvPEBP12*, *AvGRF14*, *AvFD4*, and *AvPEBP11*, demonstrated high expression specific to flowers. Cluster 2, including *AvPEBP7*, *AvPEBP10*, *AvGRF10*, *AvGRF5*, *AvGRF12*, *AvFD1*, and *AvFD2*, exhibited elevated expression in rhizomes. Cluster 3, consisting of *AvGRF11*, *AvGRF3*, *AvPEBP3*, *AvFD3*, and *AvFD5*, showed relatively high expression in flowers and P3, but low expression in other tissues. In Cluster 4, which includes *AvGRF1*, *AvGRF4*, *AvPEBP5*, *AvGRF18*, and *AvPEBP4*, *AvGRF18* was highly expressed across all tissues, with the highest level in LY3. Cluster 5, containing *AvGRF15*, *AvPEBP13*, *AvGRF7*, and *AvPEBP2*, displayed relatively high expression in P2 and P3. Lastly, Cluster 6, comprising *AvGRF17*, *AvGRF6*, *AvGRF8*, *AvGRF16*, *AvGRF2*, *AvGRF19*, *AvGRF13*, and *AvPEBP6*, showed that *AvGRF6* had extremely high expression across all tissues, while *AvGRF16* was highly expressed in P1. Overall, the clustering results from the heatmap were consistent with the differential expression analysis.

### 2.6. PPI Characteristics of FD, GRF, and PEBP Family Proteins

We constructed PPI networks to examine the potential interactions among AvFD, AvGRF, and AvPEBP family members, and their associated regulators. These networks were developed using high-confidence interaction pairs, with a confidence score exceeding 800, derived from *A. thaliana* and *O. sativa* homologs via the STRING database.

#### 2.6.1. Interactions Predicted from *A. thaliana* Homologs

**Figure 8:**
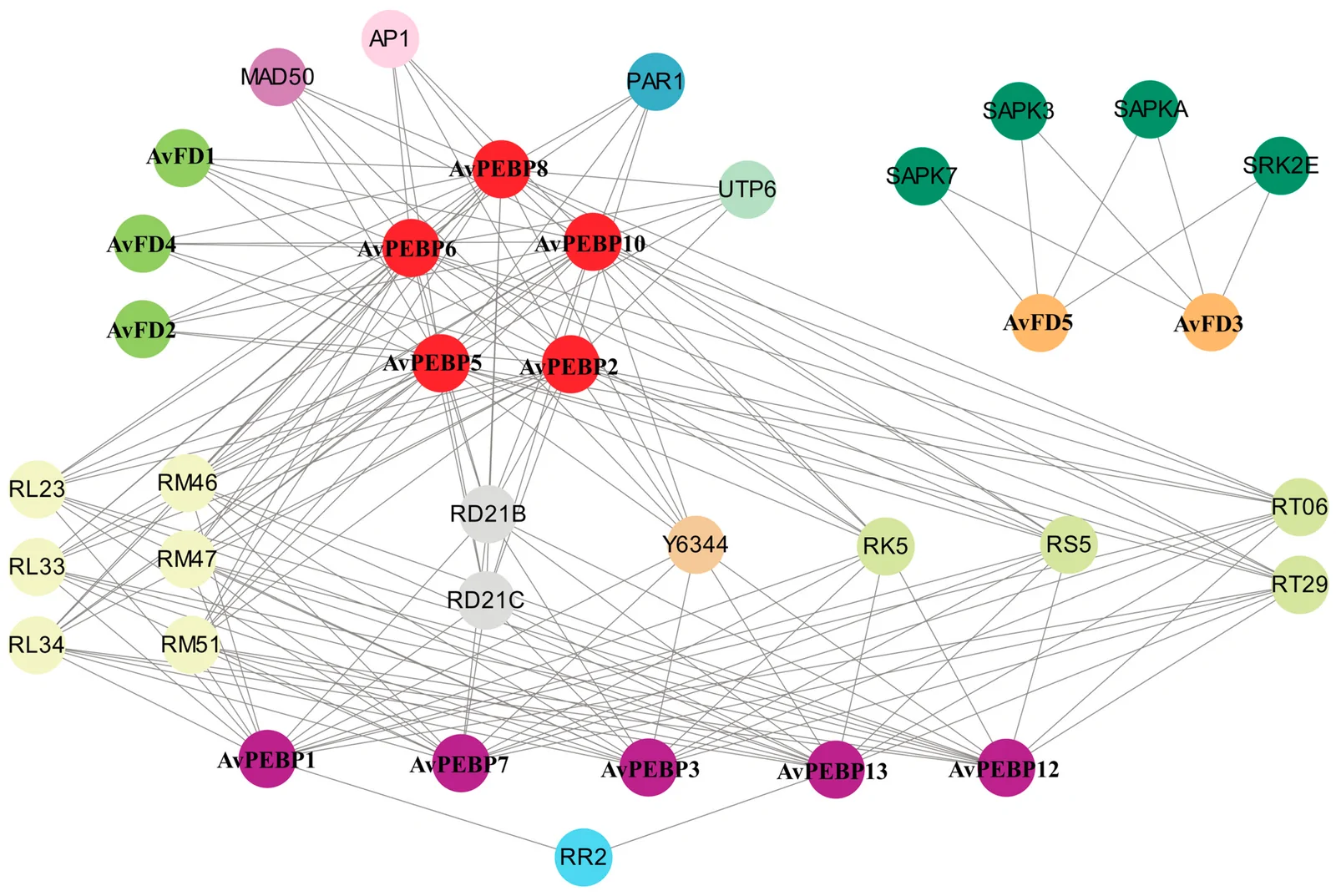
Predicted PPI network of AvPEBP, AvFD, and AvGRF proteins based on *A. thaliana* homologs.

Figure 8 illustrates that strong predicted interactions, with a score of 950, were identified between several FD-like proteins (AvFD1, AvFD2, and AvFD4) and various FT-like members (AvPEBP2, AvPEBP5, AvPEBP6, AvPEBP8, and AvPEBP10). This suggests that FD-like proteins may form stable complexes with FT-like proteins to regulate pathways related to flowering. Additionally, AvFD3 and AvFD5 demonstrated high-confidence interactions with multiple SAPK/SRK2E proteins, including SAPK3, SAPKA, SAPK7, and SRK2E, with scores ranging from 851 to 876. This implies their potential involvement in abiotic stress signaling cascades.

Comprehensive interaction profiling has demonstrated that the majority of PEBP members function as central hubs, engaging with ribosomal proteins and various regulatory proteins, as illustrated in Figure 7. These interactions included those with large subunit proteins (RL23, RL33, RL34, RM46, RM47, and RM51), small subunit proteins (RS5, RT06, RT29, and RK5), Y6344 (score = 963), ORYA (scores = 823–834), and RD21B/C (scores = 823–834). Furthermore, AvPEBP2, AvPEBP5, AvPEBP6, AvPEBP8, and AvPEBP10 displayed moderate-to-strong interactions (scores = 802–821) with AP1, PAR1, MAD50, and UTP6, indicating their potential roles in developmental or RNA processing regulation.

The predictions derived from *Arabidopsis* suggest that PEBP members serve as versatile hubs within FD-FT (flowering), FD-SAPK (stress), and PEBP-ribosomal (translation) modules, although experimental validation is still required.

#### 2.6.2. Interactions Predicted from *O. sativa* Homologs

**Figure 9:**
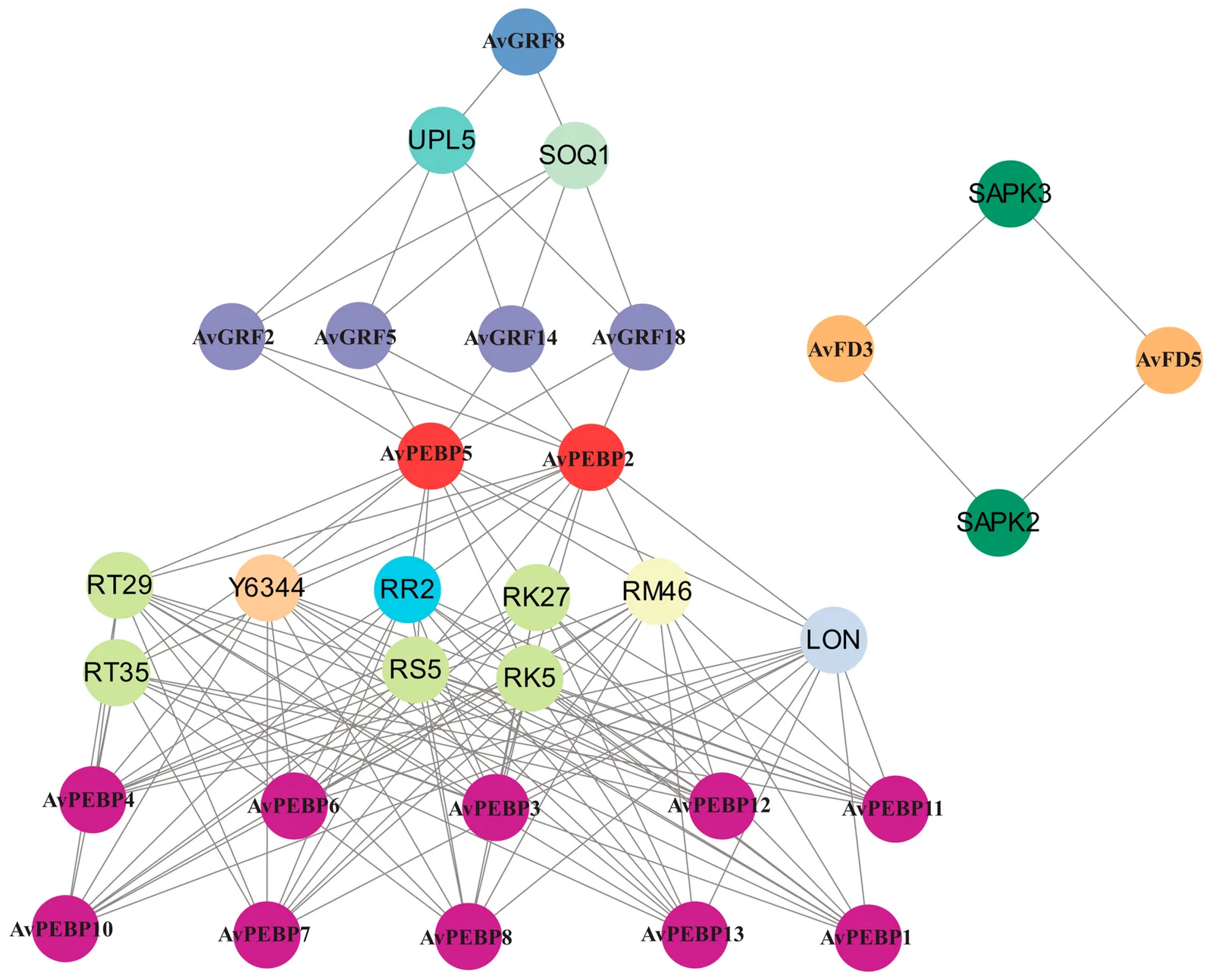
Predicted PPI network of AvPEBP, AvFD, and AvGRF proteins based on *O. sativa* homologs.

To enhance the prediction based on *Arabidopsis*, we developed a PPI network utilizing high-confidence interaction pairs (score > 800) identified from *O.sativa* homologs via the STRING database (Figure 9).

As depicted in Figure 9, significant predicted interactions (score = 997) were identified among several GRF members (AvGRF2, AvGRF5, AvGRF14, and AvGRF18) and two FT-like proteins (AvPEBP2 and AvPEBP5). These GRF proteins also demonstrated high-confidence interactions with UPL5 (score = 850) and SOQ1 (score = 800), indicating their potential roles in ubiquitination and chloroplast quality control pathways. Additionally, AvGRF8 interacts with UPL5 (score = 850) and SOQ1 (score = 800), thereby further expanding this regulatory network.

In line with the findings in *Arabidopsis*, most PEBP members functioned as central hubs, engaging with a common array of ribosomal and regulatory proteins (Figure 9). Notably, AvPEBP1 through AvPEBP8 and AvPEBP10 through AvPEBP13 demonstrated significant interactions with RR2, RT29, RK27, RK5, RS5, RM46, RT35, and Y6344. Among these interactions, the association between Y6344 and all AvPEBP members was the most robust, with a score of 913, consistently observed in both rice and *Arabidopsis* datasets.

FD-SAPK interactions were corroborated in the rice dataset (Figure 9). AvFD3 and AvFD5 demonstrated high-confidence interactions with SAPK2 (score = 828) and SAPK3 (score = 864), reinforcing the conserved function of FD-like proteins in stress signaling.

The rice-based network identified additional GRF-associated interactions not present in the *Arabidopsis* dataset, underscoring the importance of employing multiple reference species for cross-species PPI prediction. These findings suggest that monocot-specific regulatory mechanisms may be more effectively captured using rice as a reference.

### 2.7. Y2H Assays Validating Pairwise Interactions Between AvGRF/AvFD and AvPEBP Proteins

**Figure 10:**
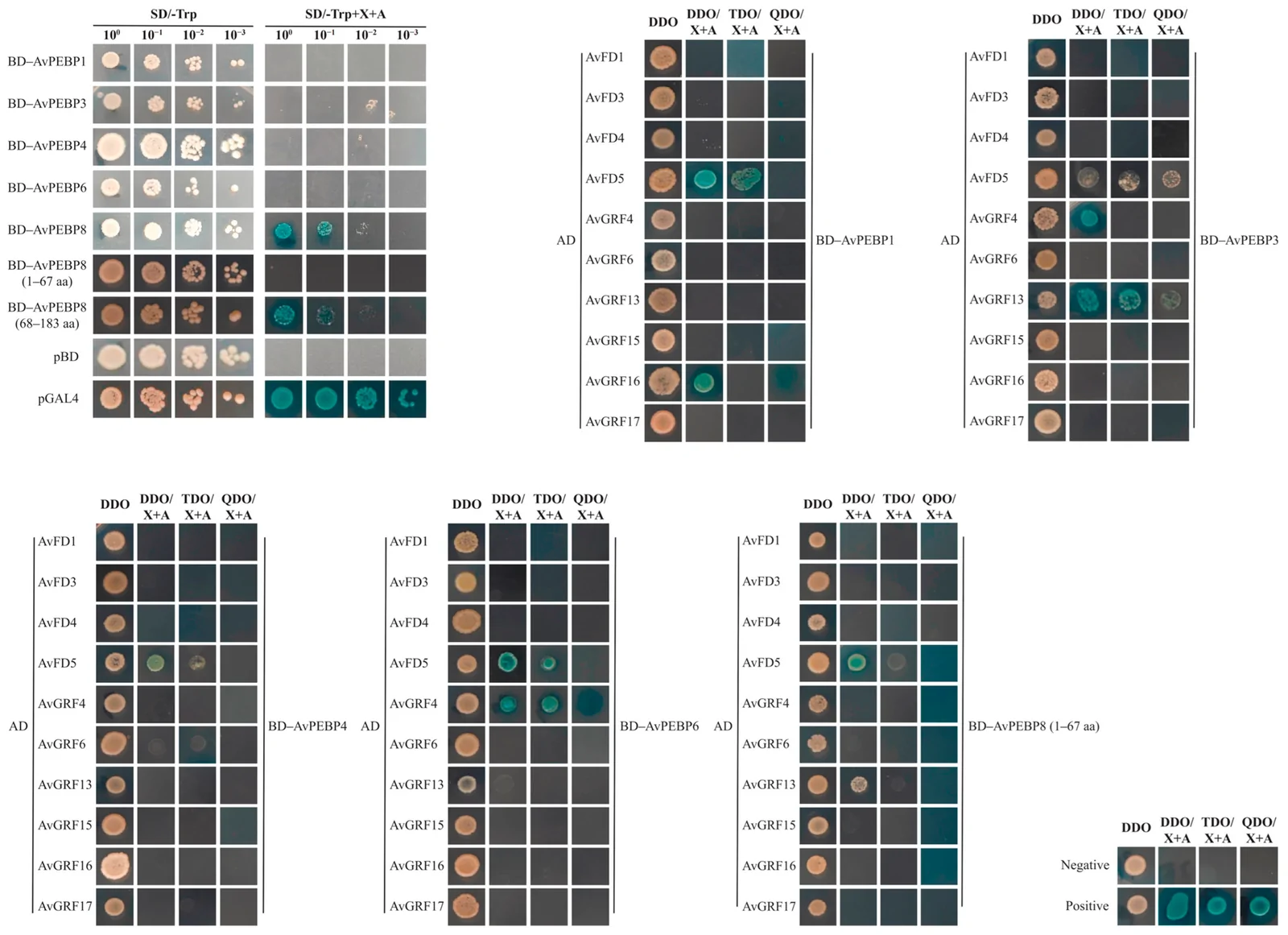
Yeast two-hybrid assays validating pairwise interactions of AvGRF–AvPEBP and AvFD–AvPEBP. DDO: SD/-Trp/-Leu (transformation control); DDO + X + A: DDO supplemented with X-α-Gal and Aureobasidin A (AbA) for LacZ reporter detection; TDO + X + A: SD/-His/-Trp/-Leu with X-α-Gal and AbA for HIS3 reporter detection; QDO + X + A: SD/-Ade/-His/-Trp/-Leu with X-α-Gal and AbA for ADE2 reporter detection.

To empirically confirm the protein–protein interactions predicted by cross-species PPI analysis, Y2H assays were conducted to evaluate pairwise interactions among selected AvGRF/AvFD and AvPEBP proteins (Figure 10). The study involved six AvGRF members (AvGRF4, AvGRF6, AvGRF13, AvGRF15, AvGRF16, and AvGRF17), four AvFD members (AvFD1, AvFD3, AvFD4, and AvFD5), and five AvPEBP members (AvPEBP1, AvPEBP3, AvPEBP4, AvPEBP6, and AvPEBP8) to examine AvGRF–AvPEBP and AvFD–AvPEBP interactions.

Transactivation assays demonstrated that none of the selected AvPEBPs exhibited transactivation activity, except for AvEPBP8. In particular, transactivation activity was observed at the C terminus (68–183 aa) of AvPEBP8 (Figure 9). Consequently, further interaction assays were conducted between the N-terminus (1–67 aa) of AvPEBP8 and other AvFDs and AvGRFs. Among the combinations tested, only a few showed positive interactions. The most robust interaction was identified between AvGRF13 and AvPEBP3, which exhibited growth on DDO + X + A, TDO + X + A, and QDO + X + A media, indicating the activation of all three reporter genes: *HIS3*, *ADE2*, and *lacZ*. The AvGRF4–AvPEBP6 pairing grew on DDO + X + A and TDO + X + A media, with blue color development on both, but did not grow on QDO + X + A medium. The AvGRF16–AvPEBP1 interaction exhibited growth and blue color solely on DDO + X + A medium, with no growth observed under higher stringency conditions. All other GRF–PEBP combinations tested, including those involving AvGRF6, AvGRF15, AvGRF17, and additional pairings with AvPEBP members, did not grow on selective media, indicating the absence of detectable interactions under the assay conditions.

When examining the interactions between AvFD and AvPEBP proteins, AvFD5 exhibited positive interactions with several PEBP members, although the interaction strengths varied. Specifically, the combinations AvFD5–AvPEBP1 and AvFD5–AvPEBP6 demonstrated growth and blue coloration on both DDO + X + A and TDO + X + A media but failed to grow on QDO + X + A medium. Conversely, AvFD5–AvPEBP4 and AvFD5–AvPEBP8 (1–67 aa) showed growth and blue coloration exclusively on DDO + X + A medium, with no growth observed under stringent conditions. No interaction was observed between AvFD5 and AvPEBP3 on any selective medium. Furthermore, all other tested combinations, including those involving the remaining AvFD members (AvFD1, AvFD3, and AvFD4) with various AvPEBP members, as well as the other AvGRF members with AvPEBPs, did not exhibit growth on selective media, indicating the absence of detectable interactions under the assay conditions.

## 3. Discussion

The fruit-setting rate is a significant agronomic trait in the cultivation of *A. villosum*, as it directly impacts the economic returns of cultivators. Various factors can influence the fruit-setting rate, with flowering time being the most important. An inappropriate flowering time, whether too early, too late, or with an overly concentrated or dispersed flowering period, can substantially reduce the fruit-setting rate.

Studies have shown that flowering time is induced in the shoot apical meristem (SAM) of plants. In the SAM, FT proteins, which are synthesized in the leaves and transported to the SAM, interact with 14-3-3 proteins and the bZIP transcription factor FD to form the FAC, thereby inducing the expression of flowering genes [8,9,10]. As essential components of the FAC, FT, 14-3-3, and FD belong to the PEBP, GRF, and FD families, respectively. In this study, we performed a genome-wide characterization of these three gene families in *A. villosum*, which will provide a foundation for elucidating the flowering mechanism in *A. villosum*.

### 3.1. Phylogenetic Divergence, Structural Characteristics, and Functional Specialization

Phylogenetic analysis categorized the 13 AvPEBP members into three conserved clades: FT-like (comprising eight members), TFL1-like (comprising three members), and MFT-like (comprising two members) (Figure 4A). This classification aligns with the established functional divergence of the PEBP family in angiosperms [13]. The number of FT-like members in *A. villosum* (*n* = 8) was significantly greater than that in *Arabidopsis* (*n* = 2), indicating a lineage-specific expansion of FT-like genes within the Zingiberales lineage. This expansion may facilitate the precise regulation of flowering time under varying environmental conditions through functional redundancy or subfunctionalization [22]. Notably, AvPEBP10 possesses a distinctly elongated C-terminus (429 aa compared to 167–183 aa in other members) (Figure 4C), suggesting that this unique structural characteristic may impact its specialized functions. The three TFL1-like members are likely to function as floral repressors by competing with FT for FD binding, thereby forming a florigen repression complex [12]. The MFT-like clade, containing only two members, is consistent with the low copy number of *MFT* genes in *Arabidopsis* and rice [13], as well as other plants [23], suggesting a non-redundant role in seed germination and developmental transitions [24]. It should be mentioned that no significant variation in motif composition was observed among the three PEBP clades (Figure 4B). This observation suggests that functional divergence among the FT-like, TFL1-like, and MFT-like subfamilies likely results from specific amino acid substitutions within conserved motifs rather than from the presence or absence of entire motifs. This finding aligns with previous reports indicating that single amino acid changes in the PEBP domain can alter FT/TFL1 activity [25].

The five AvFD members were categorized into two primary clades: FD-like (AvFD1, AvFD2, and AvFD4) and AREB3-like (AvFD3 and AvFD5) (Figure 5A), indicating functional diversification between flowering regulation and stress responses. The FD-like clade clusters were closely associated with AtFD and retained the complete bZIP domain, identifying them as core FD factors involved in flowering in *A. villosum*. Conversely, the AREB3-like clade was associated with AtAREB3 (Figure 3B), a crucial transcription factor in ABA signaling that regulates drought and osmotic stress responses [26]. Although these proteins also possess intact bZIP domains, divergent motifs were observed across all five AvFD members (Figure 5B). Specifically, AvFD3 and AvFD5 (AREB3-like) contain motifs 1–8, whereas AvFD1/AvFD4 (FD-like) lack motifs 2, 4, 6, 7, and 8, and AvFD2 includes only motifs 1, 3, 5, and 9, indicating a more streamlined motif repertoire within the FD-like clade. This progressive reduction in motif composition likely reflects functional specialization, with the AREB3-like clade retaining the most complete motif set for stress signaling, while the FD-like and FDP-like clades have streamlined their motif repertoire for more specialized flowering-related functions. Additionally, the promoter regions of AREB3-like members are enriched with ABRE and MeJA-responsive elements, suggesting a primary role in stress responses rather than in flowering regulation. This functional dichotomy likely reflects subfunctionalization following gene duplication [20].

GRFs, as plant-specific transcription factors, are integral to leaf development, floral organ formation, seed development, and plant responses to abiotic stress [27]. In this study, the 19 AvGRF members were categorized into Group I (12 members) and Group II (seven members) (Figure 6A), in accordance with the established bipartite classification of the GRF family in plants [28]. The AvGRF family size in *A. villosum* (*n* = 19) is comparable to that in other monocots, such as rice (*n* = 15) and maize (*n* = 19) [29], as well as in several dicots, such as *Helianthus annuus* (*n* = 17) [30], indicating that this family has remained relatively stable throughout Zingiberales evolution. Notably, Group I members of the AvGRF family generally possess a complete set of Motifs 1–10, whereas Group II members exhibit variations, particularly lacking Motifs 2 or 8. This differential motif composition implies that Group I may represent the evolutionarily conserved GRF core, whereas Group II members have diverged and potentially acquired specialized regulatory functions. Chromosomal mapping revealed that the GRF family is widely distributed across 15 chromosomes (Figure 2A), suggesting that GRF genes may have experienced more frequent segmental duplication events or a longer evolutionary history of dispersal.

### 3.2. Divergent FAC Assembly Mechanisms Across Species and Implications for A. villosum

FAC, typically comprising PEBP (FT-like), 14-3-3 (GRF), and FD proteins, is a central regulatory module for flowering [8,9,10]. Extensive studies have established the classical FAC paradigm, particularly in rice, where the crystal structure revealed a heterohexameric complex in which the florigen Hd3a (OsFTL2) interacts with 14-3-3 (GF14c), which in turn recruits the bZIP transcription factor OsFD1 to activate floral identity genes [10]. This model defines 14-3-3 proteins as intracellular florigen receptors. However, recent high-resolution in situ expression and biochemical investigations in *Arabidopsis* have uncovered a remarkably distinct assembly mechanism. Gao et al. (2025) demonstrated that FT is not recruited by 14-3-3 alone; instead, FT recruitment requires the DNA-bound FD–14-3-3 complex, with the unstructured C-terminus of FT directly contacting DNA [31]. Furthermore, 14-3-3 binding to phosphorylated FD (T282) suppresses FD condensation, promotes dimerization, and enhances DNA-binding activity, thereby facilitating FAC formation [31]. These discrepancies between rice and *Arabidopsis* strongly suggest that FAC assembly mechanisms are not universally conserved but have undergone lineage-specific diversification, potentially reflecting the evolutionary divergence between monocots and eudicots.

In this study, cross-species PPI predictions combined with Y2H validation revealed several unexpected features that challenge the classical FAC model in *A. villosum*. Notably, we observed significant discrepancies between the PPI networks derived from *Arabidopsis* and rice orthologs. While *Arabidopsis*-based predictions suggested robust interactions between canonical FD-like proteins (AvFD1, AvFD2, and AvFD4) and FT-like PEBPs (score 950), rice-based predictions did not (Figure 8 and Figure 9). Strikingly, the rice-based predictions were more consistent with our Y2H results, in which no direct interactions were detected between AvFD1/AvFD4 and any of the tested PEBP members under our experimental conditions (Figure 10). Several factors may account for these inconsistencies and potential false negatives. First, our Y2H assays were performed in the absence of 14-3-3 proteins, which are essential for bridging FT and FD in rice [10] and promoting FD dimerization and DNA binding in *Arabidopsis* [31]. Second, the phosphorylation status of the conserved SAP motif in FD (e.g., T282 in *Arabidopsis* FD) is critical for 14-3-3 recruitment and FAC activity [15,31]; our in vitro Y2H system likely lacks this post-translational modification, which may preclude the detection of phosphorylation-dependent interactions. Third, recent evidence indicates that DNA binding creates interfaces required for FT recruitment to the FD–14-3-3 complex [31], an element absent from standard Y2H assays. Fourth, our screening was not exhaustive; for example, although the FD-like gene *AvPEBP8* and FD-like gene *AvFD4* are both highly expressed in flowers, their potential interaction was not detected, suggesting that additional partners or specific isoforms (e.g., particular GRFs) may be required. Future systematic Y2H screening across the entire PEBP, FD, and GRF families in *A. villosum*, complemented by BiFC, pull-down, and *in planta* genetic assays, will be essential to conclusively define the FAC components.

Despite these limitations, our Y2H assays yielded one particularly intriguing and unexpected result: the AREB3-like FD protein AvFD5, rather than the canonical FD-like proteins, interacted with multiple PEBP members. AREB3-like proteins are traditionally associated with ABA signaling and stress responses [26]. However, a recent comprehensive study by Martignago et al. (2023) demonstrated that nearly all group A bZIP transcription factors in *Arabidopsis*, including AREB3, ABF1-4, and ABI5, physically interact with FT and TFL1 in yeast [19]. Importantly, genetic evidence showed that AREB3 functions redundantly with FD to mediate FT signaling at the shoot apical meristem, and *areb3fd* double mutants exhibited significantly later flowering than *fd* single mutants, confirming a genuine role for AREB3 in floral promotion [31]. In our study, *AvFD5* was highly expressed in flowers, and its encoded protein interacted with AvPEBP8, which is also flower-abundant. This raises the intriguing possibility that AvFD5 may serve as an alternative or auxiliary FD-like factor in *A. villosum* FAC, particularly because the classical FD-like proteins (AvFD1 and AvFD4) failed to interact. Nevertheless, whether AvFD5 functions as an FAC component in vivo requires further validation, including co-immunoprecipitation from native tissues, ternary complex reconstitution, and phenotypic analysis of loss-of-function mutants.

Additionally, our cross-species PPI analysis identified several conserved interaction modules extending beyond the classic flowering networks. Notably, most AvPEBP members were predicted to function as central hubs, interacting with a consistent set of ribosomal proteins (RL23, RL33, RL34, RS5, RT29, RK5, and Y6344) and the uncharacterized UPF0235 protein Y6344 in both *Arabidopsis* and rice datasets. The interaction with Y6344, recently characterized as a thermostable nucleic acid-binding protein with a distinctive α + β fold [30], was particularly robust (scores > 900 in both species). These findings imply a previously underestimated role for PEBP proteins in translation regulation or nucleic-acid-associated processes, which may be more prominent in perennial monocots, such as *A. villosum*, than in annual model plants. This observation further supports the lineage-specific neofunctionalization of PEBP family members.

In summary, our study highlights several important limitations and raises some open questions. Current Y2H experiments are limited in scope and do not systematically assess the roles of 14-3-3 proteins, FD phosphorylation, or DNA binding, all of which have been shown to critically influence FAC assembly in model species [4,15,32]. Moreover, endogenous yeast 14-3-3 proteins may have influenced our Y2H results, either by bridging interactions or competing for binding interfaces. Moving forward, we aim to systematically clone all *PEBP*, *FD*, and *GRF* genes in *A. villosum*, integrate comprehensive spatiotemporal expression profiling with high-throughput interaction assays, and combine structural modeling with in vivo genetic and biochemical approaches. Such efforts will be essential to unravel the authentic FAC composition and regulatory logic in *A. villosum* and elucidate how this unique medicinal plant coordinates flowering time in response to environmental and developmental cues.

## 4. Materials and Methods

### 4.1. Identification and Physicochemical Property Analysis of PEBP, FD, and GRF Gene Families in A. villosum

The genome data of *A. villosum* (WVV) used in this study were sourced from our previous research [32]. Protein sequences for the PEBP, FD, and GRF families were extracted from the dicot *A. thaliana* and monocot *O. sativa* (Table S3). BLASTP (BLAST v2.14.1+) searches were conducted against the WVV protein sequences using query sequences from *A. thaliana* and *O. sativa*, applying an E-value threshold of ≤1e^−15^. HMM profiles for the conserved domains of PEBP (PF01161), FD (PF00170), and GRF (PF00244) were obtained using the standalone InterProScan software version 5.63-95.0 based on the InterPro 95.0 database [33]. HMMER version 3.0 for Windows (http://hmmer.org/, accessed on 9 January 2025) was employed to identify candidate proteins within the *A. villosum* protein dataset, with an E-value threshold of ≤1e^−15^ [34]. Sequences identified through both HMMER and BLASTP were submitted to the NCBI Conserved Domain Database (CDD) (https://www.ncbi.nlm.nih.gov/cdd, accessed on 9 January 2025) to verify the presence of the respective domains.

The ExPASy ProtParam tool (http://web.expasy.org/protparam/, accessed on 12 January 2025) [35] was employed to predict physicochemical properties, such as MW, pI, and GRAVY. Subcellular localization predictions were conducted using WoLF PSORT (https://www.genscript.com/wolf-psort.html, accessed on 12 January 2025) [36].

### 4.2. Phylogenetic Analysis

Multiple sequence alignments of the PEBP, FD, and GRF amino acid sequences (Tables S3 and S4) were performed using MAFFT v7.520 [37] with the –localpair and –maxiterate 1000 options to enhance local alignment accuracy. Following alignment, poorly aligned regions were removed using TrimAl v1.4 [34] with the parameters -gt 0.1 -cons 50 to ensure the reliability of the subsequent analyses.

To comprehensively assess the evolutionary relationships, three independent methods were employed for the phylogenetic tree construction:

Maximum Likelihood (ML) trees were reconstructed using RAxML v8.2.12 [38] under the PROTGAMMAJTT model (JTT substitution matrix with gamma-distributed rate heterogeneity across sites). Rapid bootstrap analyses were conducted with 1000 replicates using the -f a option, with random seeds fixed at -x 12345 -p 12345 to ensure reproducibility. The analyses were performed using the maximum available threads (-T $(nproc)). The ML method identifies the tree topology that maximizes the likelihood of the observed sequence data given the substitution model, and 1000 bootstrap replicates were used to assess the statistical support for each internal branch.

NJ trees were generated using IQ-TREE v2.3 [39] based on trimmed amino acid alignments. The optimal amino acid substitution model (JTT + G) was automatically determined by the built-in ModelFinder module, and the NJ tree was reconstructed under this model with 1000 bootstrap replicates (-b 1000) using the -n 0 option to skip the ML tree search. Branch support was evaluated using the ultrafast bootstrap approximation implemented in IQ-TREE.

BI trees were constructed using MrBayes v3.2.7 [40] based on the trimmed amino acid alignments. The analysis was performed under the Whelan and Goldman (WAG) amino acid substitution model (prset aamodelpr = fixed(wag)) with a gamma-distributed rate variation across sites (lset rates = gamma) [41]. The WAG model, a general empirical substitution matrix for amino acid sequences, accounts for the relative rates of amino acid replacements derived from a large-scale dataset. This model was chosen due to its widespread use in phylogenetic inference of protein sequences and its demonstrated efficacy across diverse taxonomic groups. To accommodate variable evolutionary rates among sites, gamma-distributed rate heterogeneity was applied, with the shape parameter estimated from the data [42]. Two independent MCMC runs were conducted, each with one cold chain and three heated chains (default heating parameters) for 1,000,000 generations, sampling every 1000 generations. The first 2500 sampled trees (25%) were discarded as burn-in. Convergence was assessed by ensuring that the average standard deviation of split frequencies fell below 0.01 and that potential scale reduction factors (PSRF) approached 1.00 for all parameters. A 50% majority-rule consensus tree was constructed from the post-burning samples, with branch supports represented as Bayesian posterior probabilities. All resulting phylogenetic trees were visualized and edited using FigTree v1.4.4 (http://tree.bio.ed.ac.uk/software/figtree/, accessed on 12 May 2026).

### 4.3. Chromosomal Localization and Collinearity Analysis

Using the genome sequence and general feature format (GFF) file, a chromosome distribution map for the *AvPEBP*, *AvFD*, and *AvGRF* genes was constructed using TBtools v2.008 [43]. The genome sequences of *Musa acuminata*, *Canna indica*, and *Zingiber officinale* were obtained from NCBI. Collinearity files for each species pair were generated using the One Step MCScanX program in TBtools v2.008 [43]. Collinearity analysis was visualized using the Multiple Synteny Plot tool in TBtools v2.008 [43].

Genome annotations and protein sequences for AvPEBP, AvFD, and AvGRF from *A. thaliana* were sourced from the TAIR database (https://www.arabidopsis.org/download/, accessed on 17 January 2025). The genome annotation for *Musa acuminata* was retrieved from NCBI (https://www.ncbi.nlm.nih.gov/datasets/genome/GCF_036884655.1/, accessed on 19 January 2025), as were those for *Canna indica* (https://www.ncbi.nlm.nih.gov/datasets/genome/GCA_034359265.1/, accessed on 19 January 2025) and *Zingiber officinale* (https://www.ncbi.nlm.nih.gov/datasets/genome/GCF_018446385.1/, accessed on 19 January 2025).

### 4.4. Conserved Domain and Gene Structure Analysis

The conserved domains of AvPEBP, AvFD, and AvGRF proteins were identified using NCBI’s Conserved Domain Search service (CD-Search, https://www.ncbi.nlm.nih.gov/Structure/cdd/wrpsb.cgi, accessed on 9 January 2025) [44], which employs the RPS-BLAST algorithm. All searches were performed using the default parameters. The identified conserved domains were visualized using the NCBI CDD DOmainPattern in TBtools v2.008 [43]. Additionally, conserved motifs of AvPEBP, AvFD, and AvGRF proteins were examined using the MEME online tool (http://meme-suite.org/tools/meme, accessed on 27 January 2025) [45]. The analysis was performed using protein sequences as input in classic mode, with the site distribution model set to “Zero or One Occurrence per Sequence” (ZOPS) and the maximum number of motifs to discover set to 10. All other parameters remained at their default settings, as provided by the online server. The exon–intron structures (including UTRs and CDSs) of *AvPEBP*, *AvFD*, and *AvGRF* genes were visualized using the Gene Structure View tool in TBtools v2.008 [43] based on the GFF annotation file of the *A. villosum* genome.

### 4.5. Analysis of Cis-Elements in Promoters

The 2000 bp upstream sequences of *AvPEBP*, *AvFD*, and *AvGRF* genes were extracted from the *A. villosum* genome. The cis-regulatory elements within the promoters were predicted using the online tool PlantCARE (http://bioinformatics.psb.ugent.be/webtools/plantcare/html/, accessed on 5 June 2025) [46], with default search parameters, which facilitated the identification of their types, quantities, and potential functions. For visualization, the identified cis-elements were displayed in two complementary manners. First, the positional distribution of each cis-element type along the promoter regions was visualized using the Basic Biosequence View tool in TBtools v2.008 [43]. Second, the occurrences of each cis-element type per gene promoter were tallied, and a heatmap was generated using R version 4.3.0 with the tidyverse (v2.0.0) [47] and RColorBrewer (v1.1.3) packages to display the abundance of each cis-element type across all the analyzed promoters. In the heatmap, the number of occurrences for each element–gene pair is represented by color intensity, with cell values overlaid as text labels. The color gradient was defined using a custom palette ranging from light gray (low counts) to dark red (high counts), and the *x*-axis (cis-element types) was positioned at the top to improve readability.

### 4.6. Transcriptome Data Analysis

The raw RNA-seq data of *A. villosum* (WVV) used in this study were derived from 21 biological samples in our previous research [3], representing seven sample types: flowers (F), young leaves (LY3), fruits at 10, 30, 60, and 90 days after flowering (P1-P4), and rhizomes (Rh3). The SRA accession numbers for all the samples are listed in Table S6. Raw RNA-seq reads were quality-assessed using FastQC v0.11.8 (https://www.bioinformatics.babraham.ac.uk/projects/fastqc/, accessed on 9 January 2025) and trimmed using fastp v0.23.2 [48] (https://github.com/OpenGene/fastp, accessed on 9 January 2025) to remove adapter sequences and low-quality bases. The resulting clean reads were aligned to the *A. villosum* reference genome using HISAT2 v2.2.1 [49] (https://github.com/DaehwanKimLab/hisat2/releases#release-v2.2.1, accessed on 3 January 2026) with default parameters, and the aligned reads were sorted and converted to BAM format with SAMtools v1.10 [50] (https://github.com/samtools/samtools/releases?page=3#release-1.10, accessed on 3 January 2026). Transcripts were assembled from each sample’s BAM file using StringTie v2.1.7 [51] (https://github.com/gpertea/stringtie/releases#release-v2.1.7, accessed on 3 January 2026), and the resulting GTF files from all samples were merged into a non-redundant transcript annotation via StringTie’s –merge function. Gene and transcript abundances were quantified against the merged annotation using StringTie with the -e and -A options. Count matrices and transcript sequences were then extracted from the assembled GTF files using prepDE.py and gffread v0.12.7 [52] (https://github.com/gpertea/gffread/releases#release-v0.12.7, accessed on 3 January 2026), respectively.

### 4.7. PPI Predictions

Protein–protein interactions (PPIs) among AvPEBP, AvFD, and AvGRF proteins were predicted using the PPI Predict function in TBtools v2.008 [43] via sequence similarity-based homology transfer analysis. Reference PPI data were downloaded from the STRING database v12.0 (https://cn.string-db.org/, accessed on 3 January 2026) [53] using *A. thaliana* (taxon ID: 3702; file: 3702.protein.links.full.v12.0.txt) and *O. sativa* (taxon ID: 39947; file: 39947.protein.links.full.v12.0.txt) as reference species. The complete protein sequences of *A. villosum* were used as the target query sequences.

For each predicted interaction pair, TBtools assigned a confidence rating (Excellent, Good, Fair, Average, or Poor) to each protein based on the BLAST match quality and provided a C(score) reflecting the overall confidence of the predicted interaction. A two-step filtering strategy was applied to ensure the reliability of the predicted interactions. Initially, only interaction pairs in which both interacting proteins had a confidence rating of “Good” or “Excellent” were retained. Subsequently, interactions with a C(score) ≥ 800 were selected for further analysis and network visualization. In practice, all interaction pairs passing the first filter had scores ranging from 802 to 963, all exceeding the 800 threshold. The final filtered PPI networks were visualized using Cytoscape v3.10 [54]. Since this study employed a computational prediction method rather than large-scale experimental interaction data, the reliability of the predicted interactions was ensured through the stringent filtering criteria described above, rather than traditional statistical significance testing (e.g., *p*-values).

### 4.8. Yeast Two-Hybrid Assay

PCR reactions utilized cDNAs from combined tissue samples of *A. villosum* as templates. The PCR protocol included an initial denaturation at 95 °C for 3 min, followed by 32 cycles of denaturation at 95 °C for 15 s, annealing at 64 °C for 15 s, and extension at 72 °C for 50 s, concluding with a final extension at 72 °C for 5 min. Amplified products were verified through agarose gel electrophoresis and Sanger sequencing. Homologous recombination was conducted using pGADT7 and pGBKT7 plasmid vectors linearized by EcoRI, and the resulting products were transformed into DH5α *Escherichia coli* for sequencing.

Transactivation analysis assays of AvPEBPs were conducted in the yeast strain Y2HGold utilizing the Yeastmaker Yeast Transformation System 2 (Takara, Tokyo, Japan). In the Y2H assays, plasmids containing the accurate sequences of AvPEBP, AvFD, or AvGRF were introduced into Y187 or Y2HGold yeast competent cells and subsequently plated on SD/-Leu and SD/-Trp solid media. Yeast mating was performed, and the potential co-transformants were screened on SD/-Trp/-Leu (DDO) selective solid medium, followed by verification via PCR. To further confirm protein–protein interactions, SD/-Ade/-Trp/-Leu (TDO) + X + A and SD/-His/-Ade/-Trp/-Leu (QDO) + X + A media were employed. The pGBKT7-53 + pGADT7-T pair served as a positive control, while the pGBKT7-Lam + pGADT7-T pair was used as a negative control. All primers used in this study are listed in Supplementary Table S4.

### 4.9. Generative AI Statement

No generative artificial intelligence (GenAI) tools were used in the design, execution, or writing of this study. Grammatical and formatting edits were performed manually or using standard word-processing software without the use of GenAI.

## 5. Conclusions

This study conducted the inaugural genome-wide identification of the *PEBP*, *FD*, and *GRF* gene families in a Zingiberaceae species, identifying 13 AvPEBP, 5 AvFD, and 19 AvGRF members in *A. villosum*. Phylogenetic analysis indicated that the classification of the AvPEBP family into FT-like, TFL1-like, and MFT-like clades aligned with previous studies on other plant species. The five AvFD members were categorized into two major clades: FD-like and AREB3-like, demonstrating functional diversification between flowering regulation and stress responses. The GRF family, comprising 19 members, is comparable in size to those in other monocots, suggesting relative stability throughout Zingiberales evolution. Further evidence of functional divergence and evolutionary dynamics among these three families is provided by analyses of their motif composition, chromosomal distribution, collinearity, and promoter characterization.

Cross-species PPI predictions and Y2H assays have revealed that, contrary to the classical rice FAC model, where FD-like proteins primarily interact with FT-like proteins, only a limited number of FD–PEBP and GRF–PEBP combinations demonstrated detectable interactions under the tested experimental conditions. Notably, the AREB3-like protein AvFD5, unlike the FD-like proteins AvFD1 and AvFD4, interacted with multiple PEBP members, whereas the GRF protein AvGRF13 exhibited a strong direct interaction with AvPEBP3. These observations imply that FAC assembly in *A. villosum* may involve additional unknown components, specific post-translational modifications, or auxiliary factors, and that its assembly mechanism may differ from the classical rice paradigm (Hd3a/14-3-3/OsFD1). Future investigations, including co-immunoprecipitation assays, ternary complex reconstitution experiments, and systematic screening for post-translational modifications or auxiliary factors, are necessary to comprehensively elucidate the precise mechanism of FAC assembly in *A. villosum*.

Collectively, this study establishes a foundation for understanding the regulation of flowering time in *A. villosum* and provides valuable resources for future functional studies on this medicinal plant.

## Data Availability

Data is contained within the article or Supplementary Material.

## Supplementary Materials

The following supporting information can be downloaded at: https://www.mdpi.com/article/10.3390/plants15172722/s1, Figure S1: Bayesian Inference phylogenetic tree of the PEBP family members in *Amomum villosum*, *Arabidopsis thaliana*, and *Oryza sativa*. Av: *Amomum villosum*; At: *Arabidopsis thaliana*; Os: *Oryza sativa*. The gymnosperm *Ginkgo biloba* (QBG49376.1) served as the outgroup; Figure S2: Neighbor-Joining phylogenetic tree of the PEBP family members in *Amomum villosum*, *Arabidopsis thaliana*, and *Oryza sativa*. Av: *Amomum villosum*; At: *Arabidopsis thaliana*; Os: *Oryza sativa*. The gymnosperm *Ginkgo biloba* (QBG49376.1) served as the outgroup; Figure S3: Bayesian Inference phylogenetic tree of the FD family members in *Amomum villosum*, *Arabidopsis thaliana*, and *Oryza sativa*. Av: *Amomum villosum*; At: *Arabidopsis thaliana*; Os: *Oryza sativa*. The AtbZIP68 and AtbZIP16 sequences were utilized as outgroups; Figure S4: Neighbor-Joining phylogenetic tree of the FD family members in *Amomum villosum*, *Arabidopsis thaliana*, and *Oryza sativa*. Av: *Amomum villosum*; At: *Arabidopsis thaliana*; Os: *Oryza sativa*. The AtbZIP68 and AtbZIP16 sequences were utilized as outgroups; Figure S5: Bayesian Inference phylogenetic tree of the GRF family members in *Amomum villosum*, *Arabidopsis thaliana*, and *Oryza sativa*. Av: *Amomum villosum*; At: *Arabidopsis thaliana*; Os: *Oryza sativa*. The 14-3-3 protein from *Amborella trichopoda* (GF14epsilon) served as the outgroup; Figure S6: Neighbor-Joining phylogenetic tree of the GRF family members in *Amomum villosum*, *Arabidopsis thaliana*, and *Oryza sativa*. Av: *Amomum villosum*; At: *Arabidopsis thaliana*; Os: *Oryza sativa*. The 14-3-3 protein from *Amborella trichopoda* (GF14epsilon) served as the outgroup; Table S1: Physicochemical properties of PEBP, FD and GRF family members of *A. villosum*; Table S2: The cis-acting elements identified in *AvPEBP*, *AvFD* and *AvGRF* promoters; Table S3: Sequences for phylogenetic analysis; Table S4: PEBP, FD and GRF family members identified in *Amomum villosum*; Table S5: Primers used in this study; Table S6: Sample IDs and SRA accession numbers used in this study; Table S7: Description of sample abbreviations in this study; Table S8: Read counts and TPM values of *AvPEBP*, *AvFD*, and *AvGRF* genes across seven tissues of *A. villosum*; Table S9: Summary of differentially expressed genes from the *AvPEBP*, *AvFD*, and *AvGRF* families across six tissues relative to flower in *A. villosum*.

## Abbreviations

The following abbreviations are used in this manuscript:

FAC	florigen activation complex
AF	*Amomi Fructus*
FT	FLOWERING LOCUS T
PEBP	phosphatidylethanolamine-binding protein
GRF	GENERAL REGULATORY FACTOR
TFL1	TERMINAL FLOWER 1
MFT	MOTHER OF FT AND TFL1
Y2H	yeast two-hybrid
aa	amino acid
pI	isoelectric point
MW	molecular weight
GRAVY	Grand Average of Hydropathy
NJ	Neighbor-Joining
BI	Bayesian Inference
ABRE	abscisic acid-responsive element
ARE	anaerobic response element
LTR	low-temperature response element
GenAI	generative artificial intelligence
