# Supplementary material for: Genome-Wide Characterization of PEBP, FD, and GRF Families in Amomum villosum Lour. and Their Potential Roles in Flowering
Source: Plants (Basel). 2026 Sep 5;15(17):2722. doi: 10.3390/plants15172722 (PMC13567350; doi:10.3390/plants15172722)
Supplement: Supplementary file 1 [file plants-15-02722-s001.zip › Supplementary Figures.pdf]

Article

Genome-wide characterization of PEBP, FD, and GRF families in *Amomum villosum* Lour. and their potential roles in flowering

Ming Lei [1,2,3], Mei Qin [1,2,3], Wei Lin [1,2,3], Jun-jun He [4], Shao-fen Jian[1,2,3], Zhan-jiang Zhang [1,2,3,5], Cui Li [1,2,3,]* and Jing Wang [1,2,3,]*

[1] Guangxi Key Laboratory of Medicinal Resources Protection and Genetic Improvement, Guangxi Botanical Garden of Medicinal Plants, Nanning 530023, China; leiming@gxyyzwy.com (M.L.); qinmei20210630@163.com (M.Q.); 1029807779@qq.com (W.L.); jsfzc2011@126.com (S.J.); zzj1811@163.com (Z.Z.)
(Z.Z.)

[2] National Center for Traditional Chinese Medicine (TCM) Inheritance and Innovation, Guangxi Botanical Garden of Medicinal Plants, Nanning 530023, China

[3] Guangxi Engineering Research Center of TCM Resource Intelligent Creation, Guangxi Botanical Garden of Medicinal Plants, Nanning 530023, China

[4] South Subtropical Crops Research Institute, China Academy of Tropical Agricultural Sciences / Guangdong Dry Farming Water-saving Agricultural Engineering Technology Research Center, Zhanjiang, Guangdong 524013, China; hbj46@catas.cn (J.H.)

[5] Guangxi Key Laboratory for High-Quality Formation and Utilization of Dao-di Herbs, Guangxi Botanical Garden of Medicinal Plants, Nanning 530023, China

* Correspondence: licuicui941@163.com (C.L.); wangjing@gxyyzwy.com (J.W.)

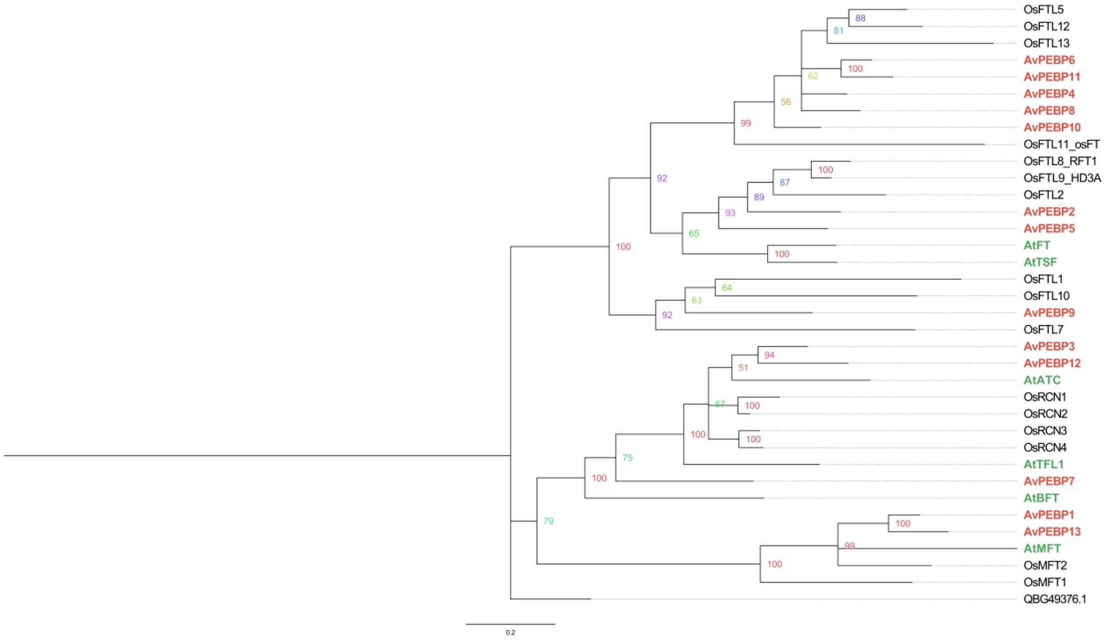

Supplementary Figure S1. Bayesian Inference phylogenetic tree of the PEBP family members in *Amomum villosum*, *Arabidopsis thaliana*, and *Oryza sativa*. Av: *Amomum villosum*; At: *Arabidopsis thaliana*; Os: *Oryza sativa.* The gymnosperm *Ginkgo biloba* (QBG49376.1) served as the outgroup.

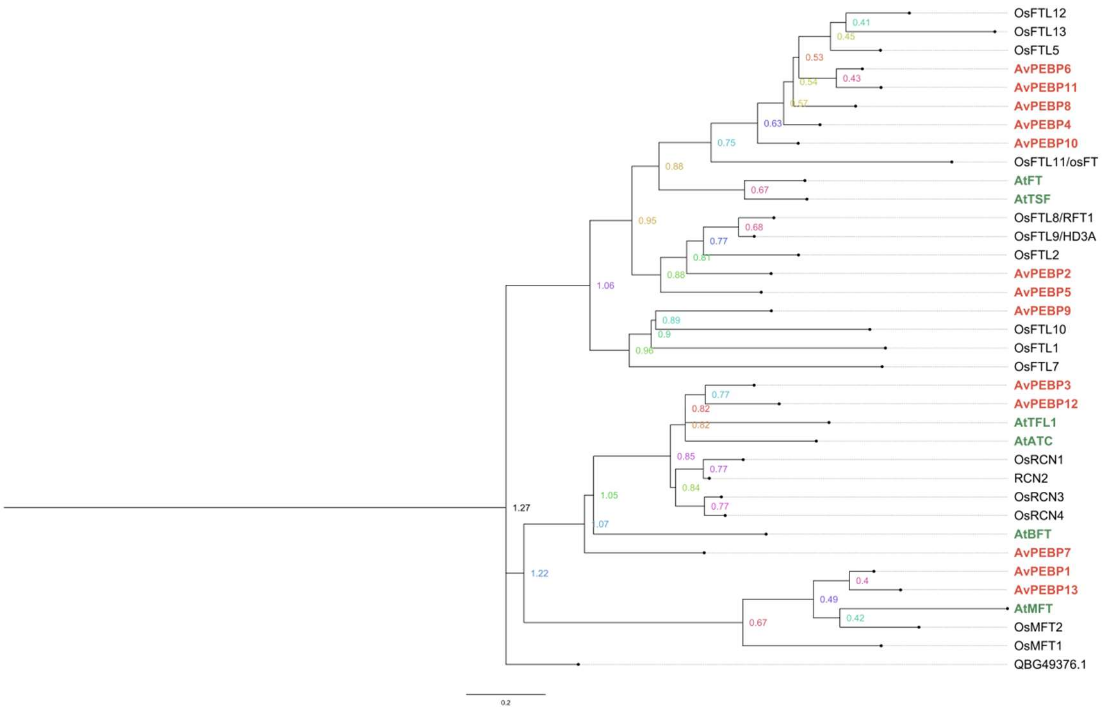

Supplementary Figure S2. Neighbor-Joining phylogenetic tree of the PEBP family members in *Amomum villosum, Arabidopsis thaliana*, and *Oryza sativa*. Av: *Amomum villosum*; At: *Arabidopsis thaliana*; Os: *Oryza sativa.* The gymnosperm *Ginkgo biloba* (QBG49376.1) served as the outgroup.

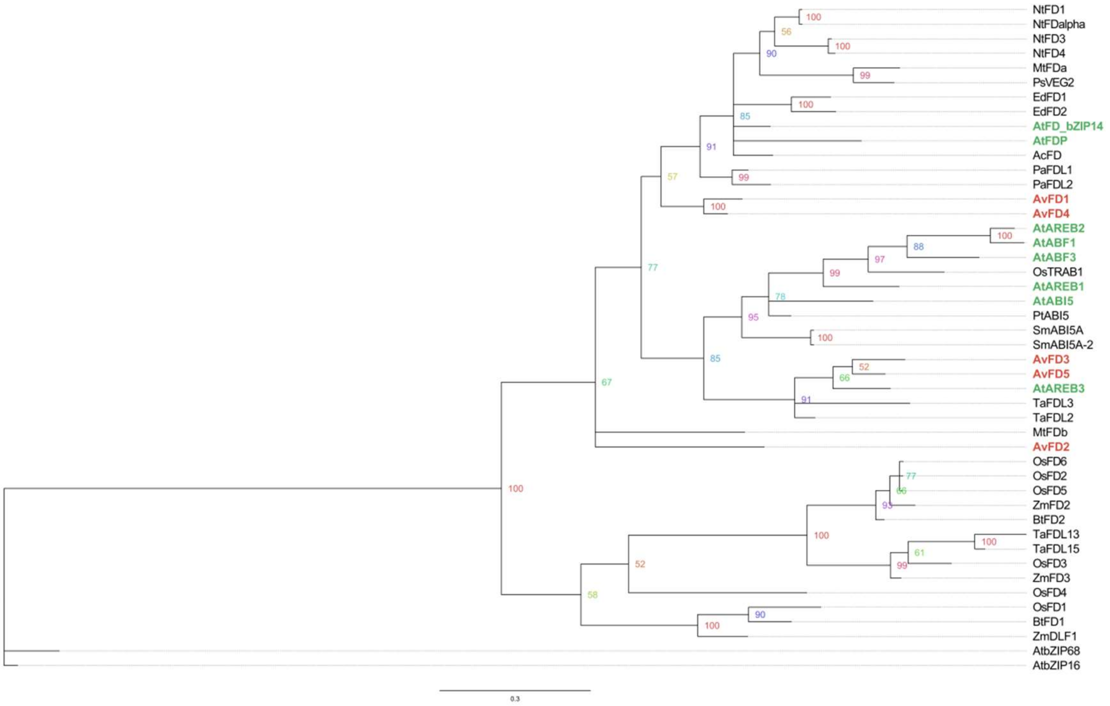

Supplementary Figure S3. Bayesian Inference phylogenetic tree of the FD family members in *Amomum villosum, Arabidopsis thaliana*, and *Oryza sativa*. Av: *Amomum villosum*; At: *Arabidopsis thaliana*; Os: *Oryza sativa.* The AtbZIP68 and AtbZIP16 sequences were utilized as outgroups.

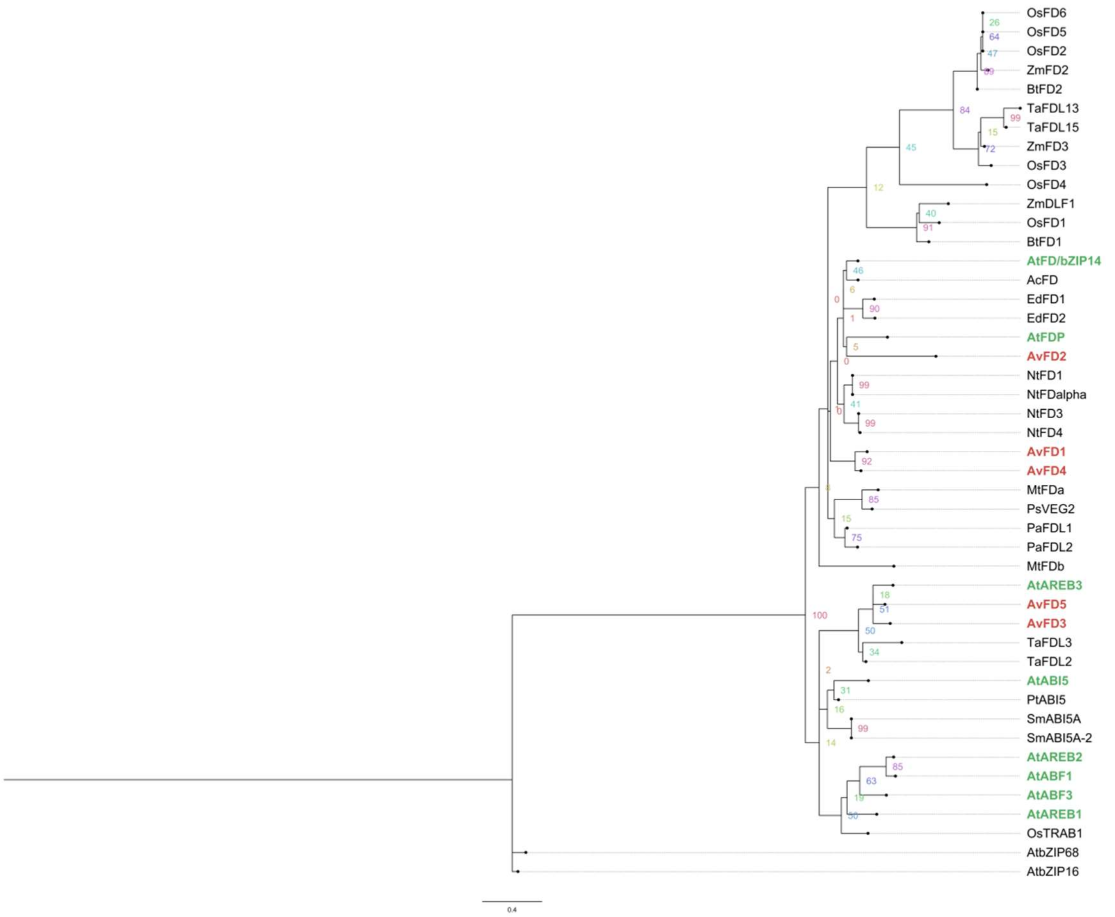

Supplementary Figure S4. Neighbor-Joining phylogenetic tree of the FD family members in *Amomum villosum*, *Arabidopsis thaliana*, and *Oryza sativa*. Av: *Amomum villosum*; At: *Arabidopsis thaliana*; Os: *Oryza sativa.* The AtbZIP68 and AtbZIP16 sequences were utilized as outgroups.

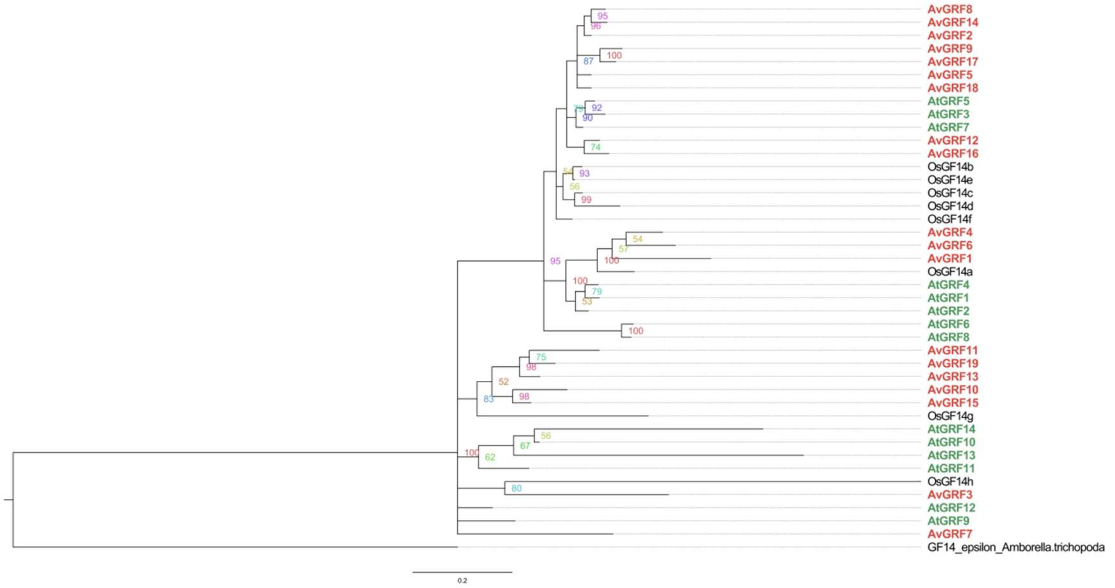

Supplementary Figure S5. Bayesian Inference phylogenetic tree of the GRF family members in *Amomum villosum*, *Arabidopsis thaliana*, and *Oryza sativa*. Av: *Amomum villosum*; At: *Arabidopsis thaliana*; Os: *Oryza sativa.* The 14-3-3 protein from *Amborella trichopoda* (GF14epsilon) served as the outgroup.

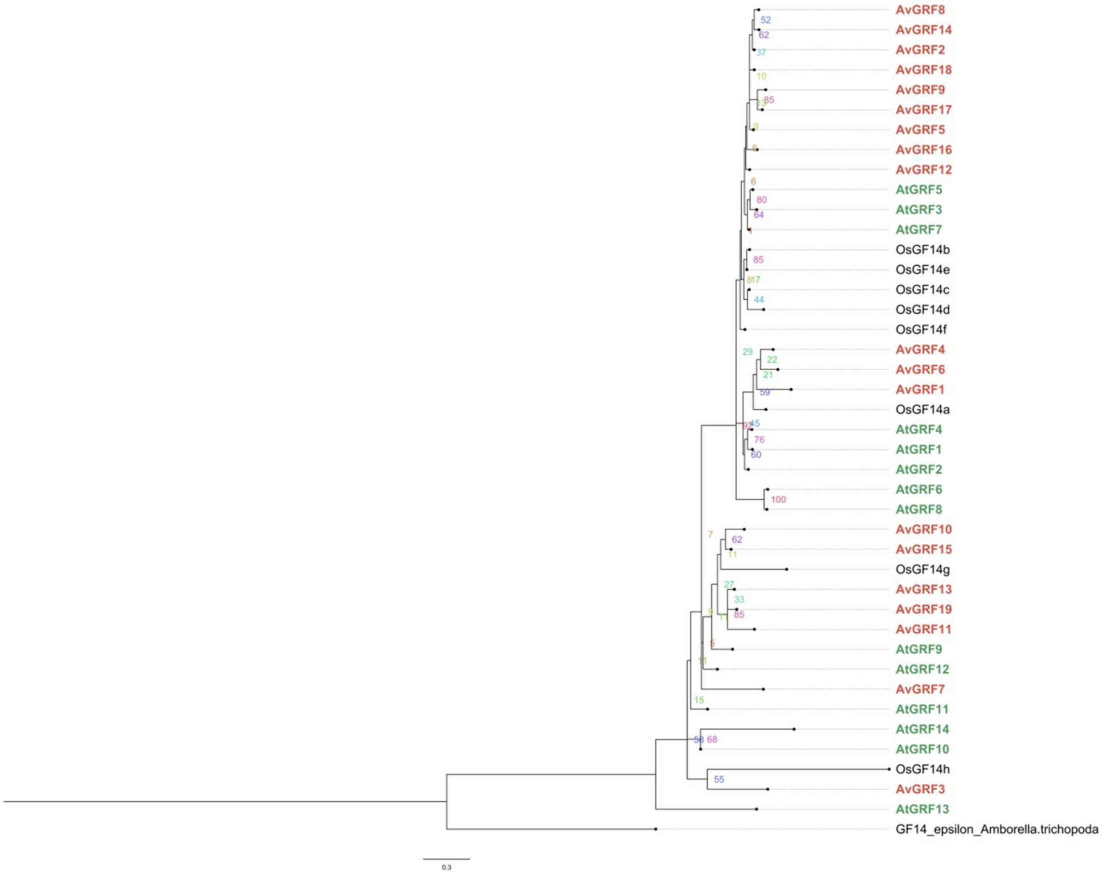

Supplementary Figure S6. Neighbor-Joining phylogenetic tree of the GRF family members in *Amomum villosum*, *Arabidopsis thaliana*, and *Oryza sativa*. Av: *Amomum villosum*; At: *Arabidopsis thaliana*; Os: *Oryza sativa.* The 14-3-3 protein from *Amborella trichopoda* (GF14epsilon) served as the outgroup.
